# Versatile Solvent‐Free Synthesis of Composite Polymer Electrolytes for Thin High‐Performance Solid‐State Lithium Metal Batteries

**DOI:** 10.1002/smll.202504166

**Published:** 2025-07-30

**Authors:** Daniel Döpping, Annika Buchheit, Xiaochen Liu, Anika Goecke, Alexander P. Grimm, Dominik Voll, Manfred Wilhelm, Martin Finsterbusch, Martin Winter, Gunther Brunklaus, Patrick Théato

**Affiliations:** ^1^ Institute for Chemical Technology and Polymer Chemistry (ITCP) Karlsruhe Institute of Technology (KIT) Engesserstraße 18 D‐76131 Karlsruhe Germany; ^2^ Institute of Energy Materials and Devices (IMD‐4: Helmholtz‐Institute Münster, Ionics in Energy Storage) Forschungszentrum Jülich GmbH Corrensstraße 48 D‐48149 Münster Germany; ^3^ Institute of Energy Materials and Devices (IMD‐2: Materials Synthesis and Processing) Forschungszentrum Jülich GmbH Wilhelm‐Johnen‐Straße D‐52425 Jülich Germany; ^4^ MEET Battery Research Center, Institute of Physical Chemistry University of Münster Corrensstraße 46 D‐48149 Münster Germany; ^5^ Institute for Biological Interfaces III (IBG‐3: Soft Matter Synthesis Laboratory) Karlsruhe Institute of Technology (KIT) Hermann‐von‐Helmholtz‐Platz 1 D‐76344 Eggenstein‐Leopoldshafen Germany

**Keywords:** CPE, hybrid electrolyte, LLZO, PEO, solid‐state battery, thin film preparation

## Abstract

The development of high‐performance solid‐state lithium metal batteries (SSB**s**) relies on the invention of efficient composite polymer electrolytes (CPE**s**) that offer both high ionic conductivity and mechanical stability. However, mixing polymers and inorganic particles often leads to inhomogeneous distributions, inhibiting ion movement. This work introduces a novel solvent‐free synthesis for thin CPE films, enabling scalable and straightforward electrolyte fabrication. The proposed hybrid electrolyte system consists of a self‐crosslinking polyether matrix incorporating lithium‐ion‐conducting ceramic particles. The synthesis method facilitates homogeneous dispersion of Li_6.45_Al_0.05_La_3_Zr_1.6_Ta_0.4_O_12_ (LLZO), thus preventing agglomeration and affording consistent electrochemical performance with film thicknesses of ≈30 µm. The ability to mix polymers and incorporate additives further boosts the electrolyte's tunability, providing a versatile approach. Electrochemical characterization reveals that the fabricated hybrid CPEs exhibit superior ionic conductivity (0.27 mS cm^−1^ at 60 °C) and compatibility with lithium metal, while their implementation in high‐mass‐loading lithium iron phosphate (LFP, 7 mg cm^2^) cathodes demonstrates exceptional cycling performance of over 200 cycles at 80% state of health (SOH) at 0.25 C. The CPEs are characterized by small amplitude oscillatory shear (SAOS) in the linear regime, Young module, tensile strength, scanning electron microscopy (SEM), energy‐dispersive X‐ray spectroscopy (EDX), and electrochemically in Li||Li and NMC_622_ (LiNi_0.6_Mn_0.2_Co_0.2_O_2_) /LFP||Li cells.

## Graphical abstract

Composite polymer electrolytes (CPEs) are manufactured by roll‐to‐roll pressing with self‐crosslinking polyethers in a solvent‐free process. CPEs exhibit uniformly dispersed Li_6.45;_Al_0.05_La_3_Zr_1.6_Ta_0.4_O_12_ (LLZO) to enable high ionic conductivity and excellent cycling with lithium iron phosphate (LFP) cathodes and Li‐metal anodes. Film thicknesses of <30 µm and remarkable mechanical stability are achieved.

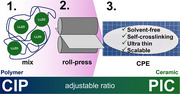

## 1 Introduction

The demand for high‐energy‐density batteries has increased significantly due to the expanding use of electric vehicles and renewable energy systems, both requiring efficient electrical energy storage systems.^[^ ^1^ ^]^ Traditional lithium‐ion batteries, though widely utilized, have limitations regarding safety, energy density, and cycle life due to potentially combustible liquid electrolytes and graphite anodes.^[^ ^2^ ^]^ Here, solid‐state lithium metal batteries may offer a promising alternative by delivering higher energy density and safety. This improvement is primarily due to the use of solid electrolytes, which offer better thermal stability and lower flammability than liquid electrolytes.^[^ ^3^ ^]^

A critical challenge in the development of SSBs compromises the design of electrolytes that combines mechanical stability of a solid material with the ionic conductivity and performance of conventional liquid electrolytes with affordable and scalable production processes.^[^ ^1^, ^4^ ^]^ One promising recently introduced development includes the combination of different solid‐state electrolytes like polymers with ceramic particles.^[^ ^5^, ^6^, ^7^, ^8^ ^]^ For ceramic particles there are a plethora of inactive (non‐lithium ion conducting) and active (lithium ion conducting) oxides available. Several studies reported improved ionic conductivities in mixed systems of poly(ethylene oxide) (PEO) with various inorganic particles of MoO_3_,^[^ ^9^, ^10^ ^]^ SiO_2_,^[^ ^11^, ^12^ ^]^ and Al_2_O_3_,^[^ ^13^, ^14^ ^]^ respectively, compared to pristine PEO polymer electrolytes. This is attributed mainly to the reduced crystallinity of PEO domains and formation of a Li‐ion‐conducting polymer‐particle interfaces, that boosts the ionic conductivity even further.^[^ ^15^ ^]^ Moreover, adding active particles like LLZO^[^ ^7^, ^16^ ^]^ or Li_1.5_Al_0.5_Ti_1.5_(PO_4_)_3_ (LATP) can increase the properties of CPEs with respect to ionic conductivity and especially Li^+^ transference number (*t* ^+^_Li_) beyond inactive filler materials.^[^ ^6^, ^17^, ^18^, ^19^, ^20^ ^]^

However, the preparation of CPEs is often tedious, expensive, and not readily scalable beyond laboratory scale.^[^ ^18^, ^21^, ^22^, ^23^ ^]^ Furthermore, inorganic filler tend to sediment, agglomerate, or even separate from the polymer matrix, which yields performance loss in CPEs.^[^ ^24^, ^25^, ^26^ ^]^

This work provides insight into a novel CPE system, contributing to the development of safer amd more efficient SSBs. The findings could pave the way for future improvements in battery technology and broader adoption of SSBs in electric vehicles and energy storage applications. Herein, we introduce a hybrid electrolyte system designed to optimize the electrochemical performance of SSBs by simplifying the producibility and customizability of CPEs. The proposed hybrid electrolyte is composed of a polymer matrix self‐crosslinking *via* ceramic particles in a solvent‐free process that is straightforward to replicate. The unique combination allows for a balance of mechanical strength and charge carrier transport properties. Also, the successful synthesis of electrolytes with a wide range from 25 to 400 % of filler content compared to the polymer mass illustrates the suitability of the proposed materials design to obtain thin ceramic‐in‐polymer (CIP) and polymer‐in‐ceramic (PIC) electrolyte films. We explore the structural, electrochemical, and thermal properties of the introduced hybrid electrolytes assessing their potential for application in SSBs. To validate the performance and properties of the hybrid electrolytes, we invoke a series of methods ‐ including electrochemical impedance spectroscopy (EIS), plating‐stripping experiments, and charge/discharge cycling in prototype SSB cells. The results demonstrate improved ionic conductivity, enhanced cycle life, and stability, indicating the hybrid electrolyte's potential to significantly advance polymer‐based SSB technology. In addition, we compare the hybrid electrolyte's performance with two different polymer matrices and different additives. An advantage of this procedure compromises facile modification of the membrane properties, e.g., by varying different parameters of the system, such as particle sizes, polymer species, polymer length, and/or additives, respectively.

**Figure 1:**
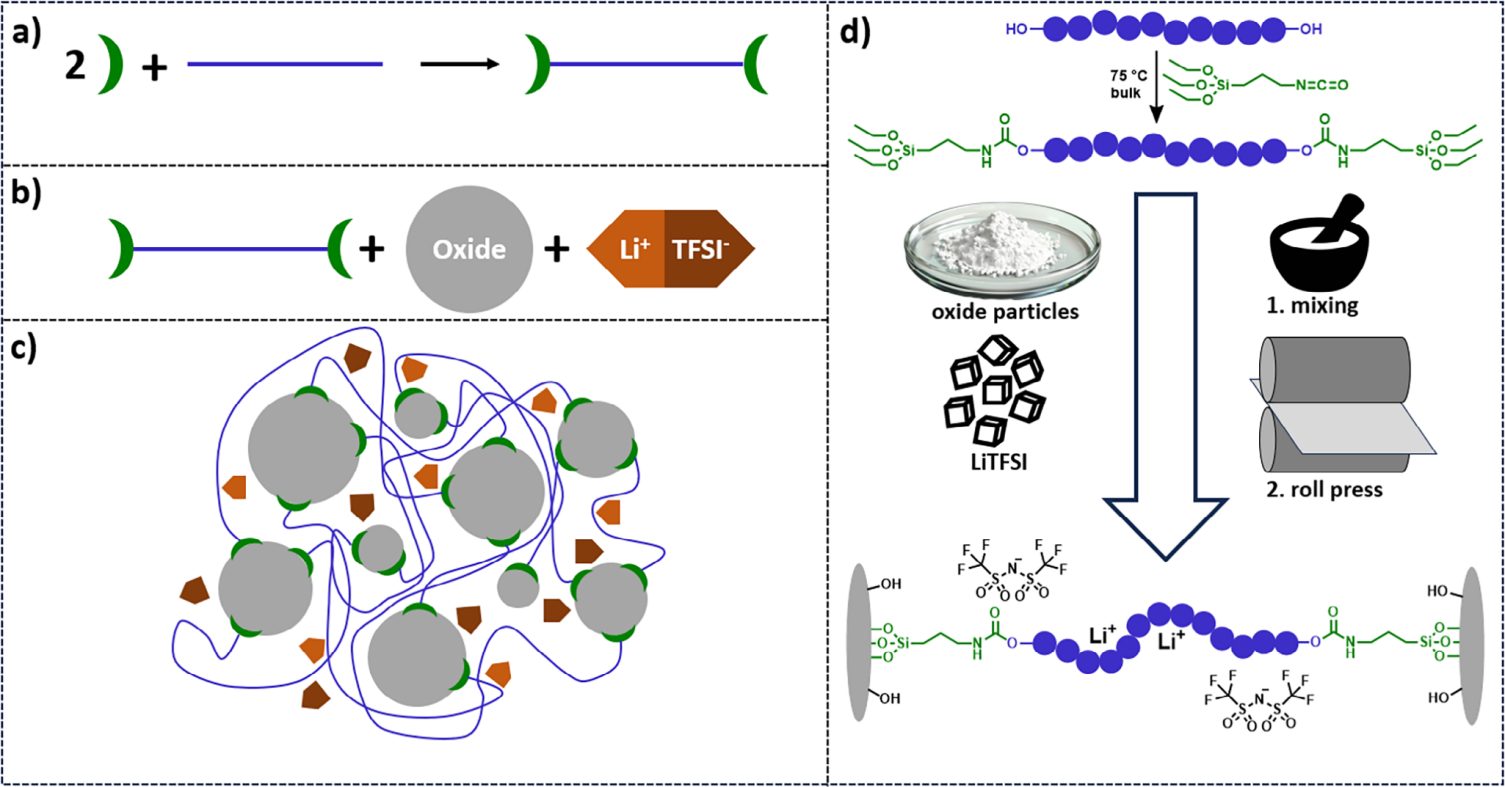
Schematic representation of the synthesis of hybrid films by a) modification of *α*,*ω*‐hydroxy‐terminated polymers (blue) with 3‐(triethoxysilyl)propyl isocyanate (IPTES, green), b) subsequent mixing with oxide particles (grey) and LiTFSI (orange) into a slurry, and c) proposed crosslinked structure of the CPE. d) Detailed overall processing scheme including highlighted chemical structures.

The schematic synthesis procedure is shown in **Figure** **1**. To obtain a self‐crosslinking system, first, *α*,*ω*‐hydroxy‐terminated polymers were modified with 3‐(triethoxysilyl)propyl isocyanate (IPTES).^[^ ^27^ ^]^ Afterward, the modified polymers were mixed with oxide particles and lithium bis‐(trifluoromethanesulfonyl) imide (LiTFSI). LiTFSI plasticizes the modified polymer and enabled the formation of a slurry out of the dry components: the silane‐modified polymer, LiTFSI, and the ceramic particles. The slurry was then roll‐pressed in between two sheets of Mylar foil (a biaxial stretched PET foil). Due to the basic or acidic nature of the ceramic particles, the alkoxy silanes began to react with free hydroxy groups at the surface of the ceramic particles. Consequently, the slurry crosslinked into a flexible membrane within 10 min to 24 h depending on the components used.

Zhang et al. used IPTES to successfully improve the cathode‐electrolyte interphase by modifying the cathode active material (CAM) with IPTES to enable an in‐situ covalently bond particle‐polymer network.^[^ ^28^ ^]^ Hence, the modified polymer could also be used as cathode binder or infiltrated into its porous structure.

In preliminary tests different polymer and ceramic particle combinations were evaluated. For polymers the requirements for consideration include suitable end‐groups for modification with IPTES, sufficient ionic conductivity, and (ideally commercial) availability. Here, two polymers were identified that matched these requirements: poly(ethylene oxide) (PEO) and polytetrahydrofuran (PTHF).

PEO is commonly used as a polymer electrolyte^[^ ^29^, ^30^, ^31^ ^]^ and one of the most studied polymers for Li‐metal batteries.^[^ ^32^, ^33^, ^34^ ^]^ PEO is commercially available in large quantities with different chain lengths, end group functionalities at affordable costs and therefore is an easily accessible polymer matrix material. Moreover, it is already exploited in the frame of solid‐state Lithium Metal Polymer technology (LMP) introduced by the Bolloré group.

In contrast, PTHF is scarcely studied as polymer electrolyte, mainly due to its low ionic conductivity and softness, which requires crosslinking to enable robust film formation.^[^ ^35^, ^36^, ^37^ ^]^ Nevertheless, PTHF shows an exceptionally high lithium transference number of 0.53 according to Mackanic et al.^[^ ^35^ ^]^ Additionally, PTHF is produced in large quantities as a commodity polymer for its use in spandex/elastan fiber production. However, due to the use as a softening agent for polymer fibers the commercially available chain lengths are limited to ≈2900 g mol^−1^.

Other polymers such as poly(*ε*‐caprolactone)‐diol (2000 g mol^−1^, BLD Pharmatech) were considered as well but no stable film formation was observed. This might be due to the relatively short chain lengths without physical entanglements of the commercially available polymers leading to a narrowly crosslinked system with high rigidity. Through the synthesis of well‐defined *α*,*ω*‐hydroxy‐terminated polyethers, polyesters, and polycarbonates for the polymer matrix many more systems are in principle conceivable. However, this was not part of this study, as the primary goal is to provide a readily accessible platform for modification and optimization that does not require expertise in controlled polymerization techniques. Hence, solely affordable and commercially available polymers were investigated subsequently.

A key factor of this study is that the polymer modification was conducted in bulk without the addition of solvents. This method has the advantage of not requiring any work‐up. The successful modification of the polymers, PEO and PTHF, with IPTES was confirmed via SEC, ^1^H NMR, and IR spectroscopy (refer to Supporting Information, Synthesis).

As active ceramic particles LATP and LLZO were exploited, though no stable film formation could be observed for LATP. An unconfirmed hypothesis suggests that LATP exhibits only a small number of hydroxy groups at its surface, which would drastically reduce its capability to act as crosslinker. Preliminary attempts with methanol/HCl activated Al_2_O_3_ particles indicated that the actual degree of crosslinking is highly dependent on the surface reactivity of particles incorporated. Therefore, the more surface reactive LLZO was selected for its reproducible and straight‐forward film forming properties. The synthesis of LLZO is described in the Supporting Information.

## 2 Results and Discussion

To discern the different films and their components in the following, films are labeled as follows: POLYMER_[O]:[Li]_LLZO %. The LLZO % is calculated by $\frac{m(LLZO)}{m(polymer)}*100$ with *m* being the mass of the respective components.

For PTHF there is no reliable information on the [O]:[Li] ratio for optimal ionic conductivity in a CPE system containing LLZO. Hence, in a first evaluation PTHF and LLZO are mixed 1:1 by weight with different [O]:[Li] ratios (5:1, 10:1, 15:1) to identify the optimal ratio. While the ionic conductivity of PTHF/LLZO membranes with 3 different salt concentrations was overall very similar, an [O]:[Li] ratio of 5:1 had the highest ionic conductivity (Figure S3, Supporting Information). Hence, for all PTHF CPEs the [O]:[Li] ratio of 5:1 was used to determine the amount of LiTFSI.

PEO/LLZO and PEO‐like/LLZO hybrid membranes were already studied in different experimental^[^ ^6^, ^17^, ^38^ ^]^ and simulated^[^ ^39^, ^40^ ^]^ setups. For PEO/LLZO CPEs an [O]:[Li] ratio of 15:1 performed best in simulated and experimental studies, which was consequently used to prepare all of the following PEO/LLZO CPEs.

### 2.1 Mechanical and Rheological Properties

Measuring tensile strength and shear rheology of hybrid polymer electrolytes is essential for their industrial application as the mechanical properties have to be within a certain range for both processing and later usage. Shear rheology can provide valuable insight into the resistance to dendrite growth and therefore cycling stability, while tensile strength ensures mechanical integrity under stress, during processing, and later application.^[^ ^4^ ^]^ Hence, both of these properties were analyzed for PTHF and PEO films incorporating 25, 50, 100, and 200 % LLZO. All films were prepared with a thickness of 200 µm. Graphs of rheological measurements can be found in Figure S4 (Supporting Information).

#### 2.1.1 Shear Rheology

**Figure 2:**
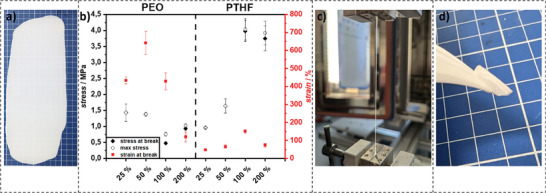
a) Example of a PEO‐LLZO film after roll‐to‐roll pressing (size of blue squares: 1x1 cm. b) Tensile strength and strain at break of PTHF and PEO with 25, 50, 100, and 200 % LLZO. c) Visual representation of the high stretchability of PEO_15_50. d) Bending test on a punched‐out test film.

Frequency sweep tests in the linear regime at room temperature demonstrated that the storage modulus (G′) was consistently higher than the loss modulus (G″), confirming the dominantly elastic behavior of the composites at all investigated frequencies (0.1–100 rad·s^−1^) (refer to Figure S4, Supporting Information). Notably, the phase angle (δ) remained below 30 degrees phase shift across all frequencies, indicating elastic behavior for all samples, and was visually confirmed by bending tests (refer to **Figure** **2**d). The storage modulus at an angular frequency of 100 rad·s^−1^ was determined for all samples. Interestingly, G′ does not have a linear correlation with the LLZO content (Figure 2b). PEO_15_50 had the lowest storage modulus with 0.3·10^5^ Pa, while PEO_15_25 and PEO_15_100 showed an equal G′ of 0.6·10^5^ Pa. PEO_15_200 exhibited expectedly the highest G′ at 1.7·10^5^ Pa. On the other hand, for PTHF_5_200, the lowest G′ of 2.5·10^4^ Pa was found. PTHF_5_100 showed a slightly higher G′ of 2.8·10^4^ Pa, and for PTHF_5_25 G′ was significantly higher at 4.8·10^4^ Pa. PTHF_5_50 exhibited the highest G′ with 5.8·10^4^ Pa. One possible explanation is that the increase in G′ at seemingly arbitrary oxide content implies a maximum for shear strength that depends on silane concentration and reactive oxide particle surface area in the optimal concentration to ensure a stable crosslinked network. Since the employed PTHF had a smaller molecular weight than of PEO, the silane concentration per mass was higher. Hence, there was a maximum shear strength at a lower concentration for PTHF than there was for PEO.

#### 2.1.2 Tensile Strength

Tensile measurements of composite films were conducted to derive the mechanical properties and strength. Overall, PEO/LLZO films withstood lower stress than PTHF, but had a higher strain at break. PEO_15_25 and PEO_15_50 exhibited a maximum stress of 1.42 and 1.38 MPa, respectively. In contrast to PTHF, a further increase in the LLZO content in PEO drastically reduced the tensile strength to 0.76 MPa for PEO_15_100 and 1.02 MPa for PEO_15_200 samples. PEO_15_50 had the highest observed strain at break at ε = 640 % (Figure 2c), while PEO_15_25 failed at ɛ = 435 % strain. The strain at break for PEO_15_100 films was still high with ɛ = 430%. However, for PEO_15_200 a significant loss in flexibility was observed with a strain at break reduction to ɛ = 120 %. This shows the accelerated breaking under stress at elevated oxide content. Both, PEO_15_100 and PEO_15_200 exhibited necking behavior.

As anticipated, the tensile strength of PTHF/LLZO films improved with increasing oxide concentration, peaking at a formulation containing PTHF_5_100, which achieved a tensile strength of up to σ = 4.04 MPa. This enhancement can be attributed to reinforcing effects of oxide particles, which likely contribute to stress transfer within the polymer matrix. However, beyond this concentration, a decrease in tensile strength was observed, suggesting a potential overloading of the polymer network or poor dispersion of the oxide particles, leading to microstructural weaknesses. For PTHF_5_200 the peak stress level dropped to σ = 3.92 MPa and a slight necking was still observed. Both PTHF_5_25 and PTHF_5_50 had a significantly lower maximum tensile stress of σ = 0.96 and 1.64 MPa, respectively. The elongation at break also varied, with a maximum elongation of ɛ = 151% observed for PTHF_5_100.

The discrepancy in mechanical properties can be rationalized by the concentration mismatch between reactive surface area and alkoxysilanes between the LLZO particles and polymer end groups leading to varying crosslinking densities. In the case of PEO, the reactive surface area of the LLZO seemed to be adequately matched by silane groups, yielding in a robust covalently bound network that enhanced mechanical stability. Conversely, in PTHF, the excess silane groups may have led to a more mobile polymer network that was not adequately reinforced by LLZO particles, resulting in lower storage modulus values. This imbalance between the available silane groups and the reactive surface area of the LLZO could hinder the effective transfer of stress, thereby compromising the mechanical integrity of the composite.

The combined results from shear rheology and tensile strength tests underscore the importance of optimizing the ceramic content in CPEs. The data shows that a careful balance between polymer and oxide particle must be maintained to maximize shear strength while avoiding compromising flexibility, which is crucial for the electrolytes’ functionality in dynamic environments, such as in rechargeable batteries. Furthermore, the findings highlight the potential of tailoring the mechanical properties of polymer/LLZO electrolytes to meet specific application requirements.

### 2.2 Scanning Electron Microscopy of Films

#### 2.2.1 SEM

**Figure 3:**
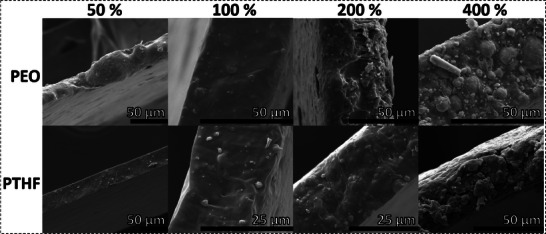
SEM micrographs of the cross‐section of PTHF/PEO LLZO films with 50, 100, 200, and 400 %. The LLZO particles are evenly distributed within the polymer and the particle density is visibly increased at higher LLZO concentrations.

The SEM analysis of the cross‐section of the composite polymer electrolyte films provided valuable insights into the morphological characteristics and distribution of the LLZO particles within the polymer matrix. First, the thickness of all considered films was between 25–65µm, indicating the suitability of the roll‐pressing methodology to achieve thin films in a reproducible manner. Second, the SEM images revealed a uniform distribution of LLZO particles throughout the polymer host, indicating effective integration and compatibility between the filler and the matrix materials (refer to **Figure** **3**).

In all examined films, LLZO particles were observed to be evenly dispersed without any significant aggregation or clustering. This homogeneous distribution is critical as it enhances the ionic conductivity and mechanical properties of the composite electrolytes. The absence of aggregates minimizes the formation of conductive pathways that could otherwise lead to ion‐blocking regions, thus improving the overall ionic transport within the electrolyte.^[^ ^25^ ^]^

Moreover, the consistent particle distribution suggested that the processing method employed ‐ roll‐pressing a high viscosity slurry ‐ was effective in also achieving a composite without sedimentation. This uniformity not only contributes to the mechanical integrity of the films but also facilitates optimal ion conduction by maximizing the contact area between the LLZO, the polymer matrix, and electrodes.

#### 2.2.2 EDX

**Figure 4:**
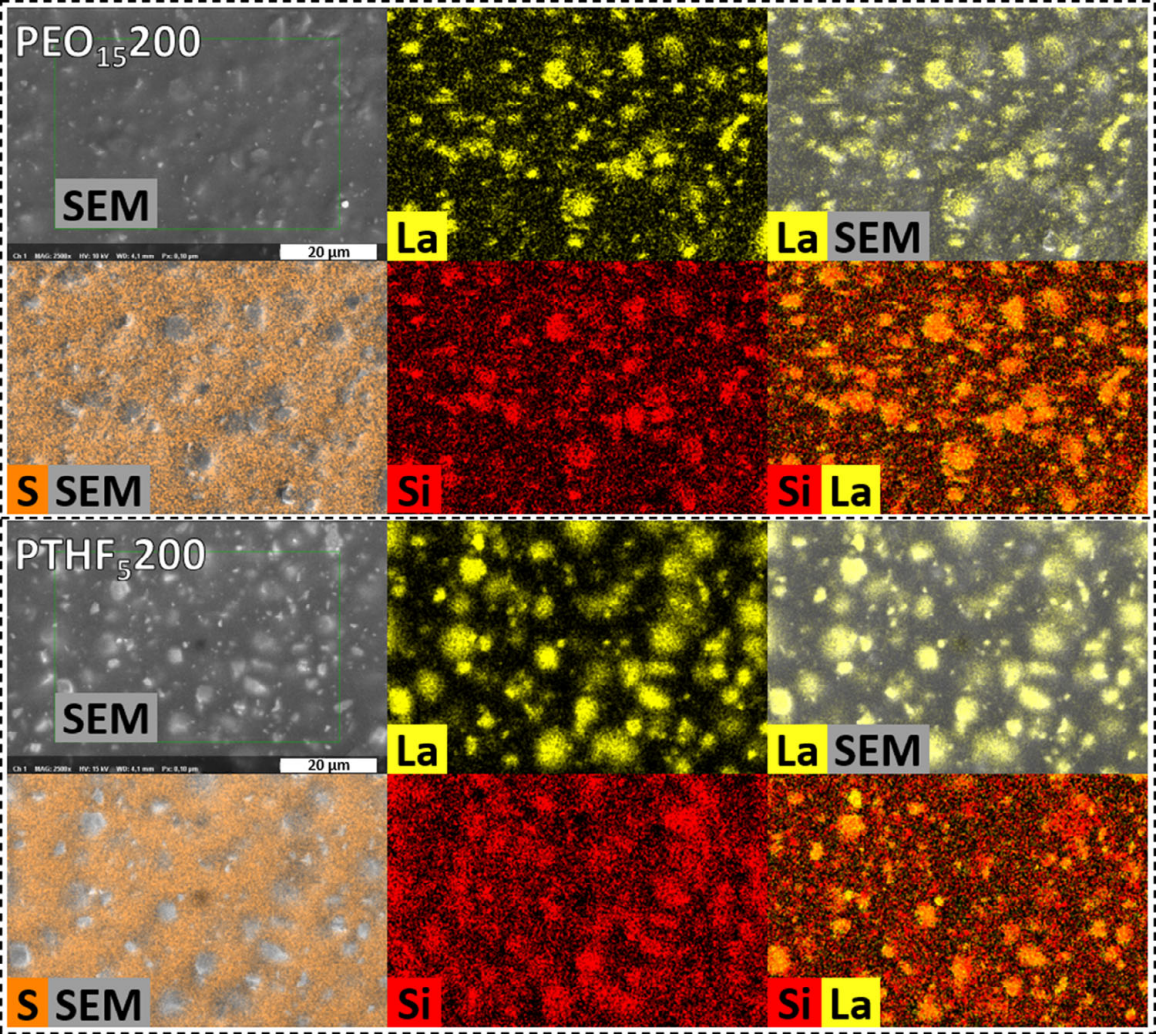
SEM of the flat surface of PEO (top) and PTHF (bottom) films with 200 % LLZO. EDX maps of the selected area. La (yellow) was chosen for identification of LLZO particles. S (orange) shows the localization of the TFSI^‐^anion. Si (red) shows the end‐groups of the respective polymer. Elemental maps and SEM micrographs are overlayed to emphasize correlation between visible LLZO particles and the La and Si maps.

The energy‐dispersive X‐ray spectroscopy (EDX) maps derived from the SEM images gave further insight into the elemental distribution within the composite polymer electrolyte films, providing critical information about the interactions between the LLZO particles and the polymer matrix (refer to **Figure** **4**). The EDX maps highlight distinct elemental contributions, facilitating a deeper understanding of the morphology and chemistry of the films.

In the EDX mapping in Figure 4, lanthanum (La) is represented in yellow, effectively confirming the localization of the LLZO particles throughout the polymer matrix. The yellow regions correspond closely to the bright areas identified in the SEM images as LLZO particles, reinforcing the notion that LLZO is uniformly distributed without aggregation. This consistent presence of La across the films emphasizes the successful incorporation of LLZO into the polymer host, which is crucial for achieving enhanced ionic conductivity. Figure 4 clearly shows the Lanthanum (La) signal, indicative of LLZO, evenly dispersed without significant aggregation. The EDX maps demonstrate that the particles are well‐separated and integrated into the matrix.

Sulfur (S), depicted in orange, corresponds to the presence of the TFSI anion and is distinctly localized away from the LLZO particles. This separation of S from the LLZO particles highlights the effective incorporation of the ionic species within the polymer matrix while maintaining a clear boundary between the LLZO and the ionic conductive pathways. The spatial differentiation of these elements suggests that the LLZO did not disrupt the ionic transport provided by the TFSI, thus preserving the electrochemical performance of the composite electrolytes.

Silicon (Si), illustrated in red, serves to localize the agglomeration of polymer chain ends. Notably, the EDX maps reveal a pronounced concentration of Si at the surfaces of the LLZO particles as indicated by the overlap of La and Si. This observation supported the hypothesis that the silane coupling agents interact predominantly at the particle surface, facilitating enhanced adhesion between the LLZO and the polymer matrix. The high concentration of Si in proximity to the LLZO particles not only confirmed this interaction but also suggested that these localized polymer segments may contribute to improved mechanical properties and ionic conductivity by creating a favorable environment for ion transport.

An intriguing observation from the EDX maps included the higher concentration of Si detected in the regions between the LLZO particles in the PTHF‐based composite films. This suggested that PTHF had a greater number of silane groups compared to the reactive surface area available on the LLZO particles. The enhanced Si concentration in these interstitial areas implied that the silane coupling agents were effectively utilized, leading to a potential saturation of reactive sites on the LLZO surface.

This finding correlates with the rheological behavior observed in the shear rheology tests, where the PEO‐based composite exhibited the highest storage modulus at 200 % LLZO. In contrast, the PTHF composite showed the lowest storage modulus under similar conditions.

The implications of these findings are significant, as they highlight the importance of optimizing the silane content relative to the reactive surface area of the filler particles. Achieving an optimal balance can enhance both the mechanical and electrochemical performance of the composite polymer electrolytes. Further studies will be necessary to explore the impact of varying silane concentrations and their effects on the interfacial properties and overall performance.

### 2.3 Electrochemical Characterization

**Figure 5:**
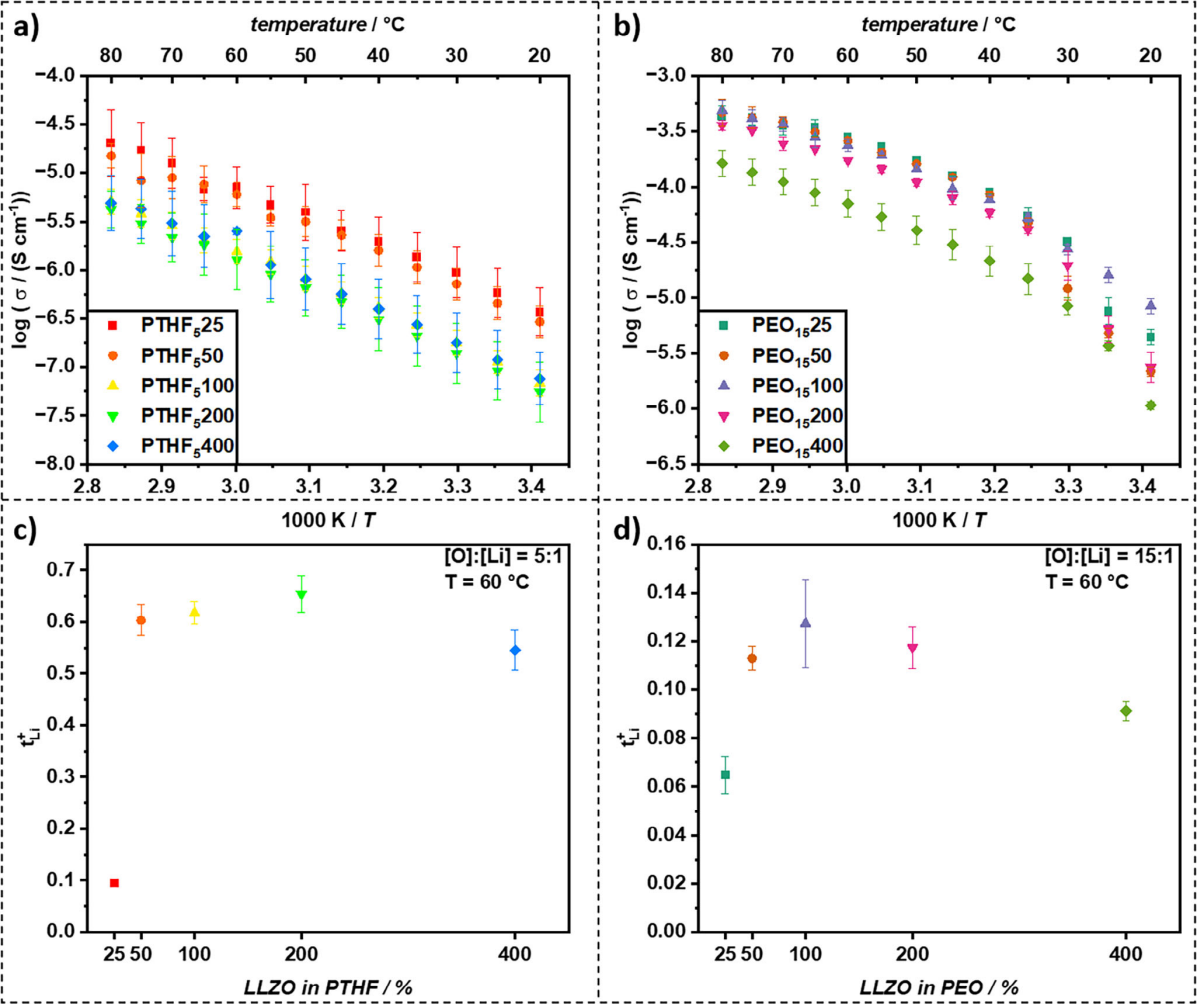
Ionic conductivity Arrhenius plots of a) PTHF and b) PEO mixed with 25, 50, 100, 200, and 400 % of LLZO from 20–80 °C. Lithium transference numbers *t* ^+^_Li_ for c) PTHF and d) PEO mixed with 25, 50, 100, 200, and 400 % of LLZO at 60 °C and a [O]:[Li] ratio of 5:1 for PTHF and 15:1 for PEO.

To get insights into the electrochemical performance, CPEs of PTHF and PEO with LLZO ratios of 25, 50, 100, 200, and 400 % were prepared to identify the impact of the LLZO content on the ionic conductivity *σ* and lithium transference number *t^+^_Li_* in a wide range (refer to **Figure** **5**).

For PTHF films, the ionic conductivity with PTHF_5_25 and PTHF_5_50 was highest with 7.06 and 6.02 µS cm^−1^ at 60 °C, respectively. Adding more LLZO let the ionic conductivity significantly drop to 1.55, 1.28, and 2.52 µS cm^−1^ for 100, 200, and 400 % at 60 °C. It should be noted that *σ* of PTHF_5_400 at 60 °C seems to be an outlier with a higher ionic conductivity than to be expected from a linear extrapolation from the values at other temperatures. Overall, the ionic conductivity of PTHF_5_25 and PTHF_5_50 was highest at all measured temperatures with a clear linear trend from 20 to 80 °C. The activation energy changed in the same manner as the ionic conductivity with PTHF_5_50 exhibiting the lowest value at 0.58 eV. PTHF_5_25, PTHF_5_100, PTHF_5_200 and PTHF_5_400 showed slightly higher activation energies at 0.60, 0.62, 0.63, and 0.64 eV, respectively. The initial increase in conductivity with filler (up to 50% LLZO in PTHF) can be attributed to the formation of favorable ion conduction pathways at the polymer‐ceramic interface and potential disruption of polymer crystallinity. However, at higher LLZO loadings (100% and above in PTHF), several factors could contribute to the observed drop and subsequent plateau in conductivity like agglomeration/particle packing. While SEM shows good dispersion, at very high filler content, the likelihood of particle‐particle contact increases, which can lead to tortuous ionic pathways if the particle‐particle interface is resistive, or if these contacts disrupt the continuous polymer phase needed for ion transport. Furthermore, a dilution effect may reduce conductivity. The polymer phase is essential for ionic conduction. Excess ceramic can dilute the conductive polymer phase, reducing the overall volume fraction of the primary conducting medium. Similarly, the nature of the interface properties changes with high LLZO loading. While the silane coupling is intended to improve interfacial contact, an excessive number of interfaces or changes in polymer chain mobility near a very high concentration of particle surfaces might become detrimental. Also, higher LLZO content significantly increases slurry viscosity, potentially affecting the final film morphology and homogeneity despite the roll‐pressing method. For PTHF, with its lower molecular weight and thus higher silane concentration per mass compared to PEO, this optimum might be reached at lower ceramic loadings. The ionic conductivity of PEO films with 25, 50, and 100 % were very similar with 0.27, 0.26, and 0.24 mS cm^−1^ at 60 °C, respectively. Increasing the LLZO content to 200 % reduced the ionic conductivity slightly to 0.17 mS cm^−1^, while increasing it further to 400 % led to a significant drop to 0.07 mS cm^−1^. The PEO hybrid films had a non‐linear behavior with an elevated loss in ionic conductivity below 50 °C. This correlated to the melting points determined by differential scanning calorimetry (DSC) measurements (refer to Figures S5–S7, Supporting Information), which ranged from 40 °C to 52 °C depending on the amount of LLZO added. Due to the varying behavior, PEO_15_100 performed best at lower temperatures from 20 to 30 °C (8.44 µS cm^−1^ at 20 °C), while still being among the best performing films at higher temperatures. PEO films demonstrated overall lower activation energies compared to PTHF films with 0.29, 0.41, 0.38, 0.47, and 0.48 for PEO_15_25, PEO_15_50, PEO_15_100, PEO_15_200, and PEO_15_400, respectively.

The lithium transference number of PTHF_5_25 (*t* ^+^_Li_ = 0.09) was a significant outlier from the other measurements, which were in a range of 0.55–0.65 (Figure 5). The *t* ^+^_Li_ values obtained for higher LLZO content were similar and even slightly higher than reported in the literature. The applied Bruce‐Vincent method is a widely accepted standard for the determination of transference numbers. However, many assumptions, such as high salt dissociation and dilution, may not apply to CPEs. Hence, the obtained numbers may only used comparatively but should not be treated as absolute values. While complementary techniques like PFG‐NMR exist, they were not available for this study.

PEO_15_25 also had the lowest *t* ^+^_Li_ with 0.06. The highest *t* ^+^_Li_ value was found for PEO films with 100 % LLZO at 0.13, while films with 50, 200, and 400 % LLZO exhibited slightly lower *t* ^+^_Li_ values. For PEO‐based systems, it is known that anion mobility (TFSI^−^) can be significant.^[^ ^39^ ^]^ Our reported *t* ^+^_Li_ values for PEO‐LLZO CPEs (e.g., up to 0.13 for PEO_15_100) are higher than pristine PEO‐LiTFSI systems (which are typically lower at ≈0.1 though literature values vary widely) and indicate that LLZO does contribute to enhancing *t* ^+^_Li_, likely by interacting with anions or modifying polymer segment dynamics. The incorporation of LLZO is expected to influence *t* ^+^_Li_ by providing additional Li‐ion pathways and potentially immobilizing anions at interfaces.

Thermogravimetric analysis (TGA) showed decomposition temperatures of ≈230–330 °C for PTHF films and ≈360 °C for PEO films highlighting high temperature stability (refer to Figures S6–S8, Supporting Information).

All in all, PTHF_5_50 and PEO_15_100 displayed the highest ionic conductivity and lithium transference number for their respective polymer. The statement that these two mixtures represented the optimum polymer to LLZO ratio could be strengthened with the help of EIS measurements in symmetrical Li cells. For this purpose, an impedance spectrum of freshly prepared cells was recorded every hour and analyzed by distribution of relaxation time (DRT) analysis (see Figures S9–S14, Supporting Information). Time constants between 10^−5^ and 10^−2^ s mainly describe ion migration through interfaces^[^ ^41^, ^42^ ^]^ and are in the following referred to as interface resistance. The time required for the cells to equilibrate was reduced as the LLZO content was increased from 25 to 100 % and increased again from 100 to 400 %. The same trend was observed for the overall interface resistance, which was lowest for PTHF_5_50 and PEO_15_100.

However, for potential cell application, the actual ionic conductivity was still too low even at 60 °C for PTHF_5_50. During cell assembly, PTHF films were easier to handle and allowed for thinner films, hence it would be desirable, to increase the electrochemical performance of PTHF through mixing with PEO to combine desirable properties of both polymers.

### 2.4 PTHF Film Optimization

**Figure 6:**
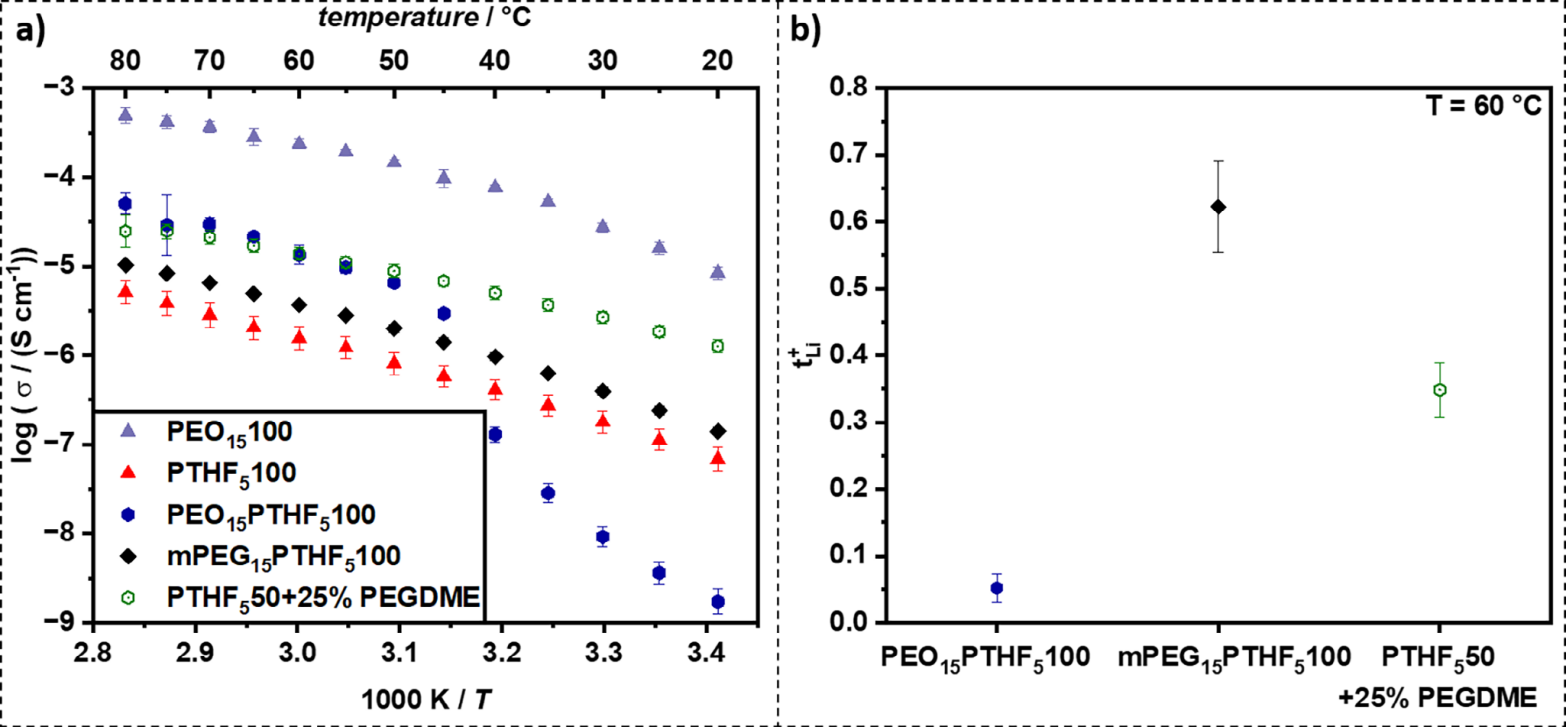
a) Ionic conductivity of PTHF/PEO mixed films from 20–80 °C. PEO_15_100 and PTHF_5_100 are added for comparison. b) Lithium transference numbers for all PTHF/PEO mixed films at 60 °C.

To assess the feasibility of mixed polymer matrices and to boost the performance of PTHF films, three different approaches to PTHF‐PEO mixtures were evaluated. First, modified PTHF and PEO were mixed 1:1 by mass with their respective equivalents of LiTFSI (PEO_15_PTHF_5_100). The same amount (by combined mass of both polymers) of LLZO was added and the resulting film evaluated for its ionic conductivity and lithium transference number (refer to **Figure** **6**).

The results indicate that incorporation of PEO into a PTHF matrix enhanced its electrochemical properties, particularly at elevated temperatures. Notably, at temperatures above 45 °C, which is above the melting point of PEO, the performance of the PTHF/PEO mixture aligned closely with the average of its pure components (*σ* = 13.4 µS cm^−1^ at 60 °C), suggesting a potential synergistic effect. However, below 40 °C, the blend demonstrated inferior performance compared to both PTHF and PEO alone. The lithium transference number of 0.05 at 60 °C was also significantly lower than PEO_15_100 and PTHF_5_100 individually.

A hypothetical explanation could be that the lower glass transition temperature and lower molecular weight of PTHF enable PTHF to move more freely in the slurry, allowing it to interact with the particle surface before PEO does. Additionally, PEO is partially crystallized at room temperature hindering its diffusion. Hence, the faster surface reaction of PTHF may impair PEO's ability to react effectively with the LLZO surface, yielding “free” PEO encapsulated in a PTHF matrix. Note that two distinct transport processes can be identified in the DRT spectrum between 10^−5^ and 10^−4^ s (refer to Figures S15–S17, Supporting Information) for the PTHF/PEO mixture, evidencing competing transport paths through the sample. These findings support the above hypothesis. Additionally, below 45 °C compartmentalized PEO crystallized, which was identified via DSC (refer to Figure S7, Supporting Information) in three small separate melting points at 42.5, 30.5, and 16.7 °C. Crystallization of PEO leads to a loss in free volume of the polymer, which is the main contributor to the ionic conductivity.^[^ ^43^ ^]^ The steady crystallization of PEO from 42.5–16.7 °C aligned with the reported sharp loss in ionic conductivity even below the values obtained for pure PTHF_5_100. These findings clearly showed that the partial crystallization of PEO in the host matrix of PTHF also drastically influences the ionic conductivity of PTHF below the melting point of PEO leading to a complex interplay of temperature dependent phase interactions between PTHF, PEO, and LLZO particles.

To further explore the properties of mixed systems, mono functionalized PEO (poly(ethylene glycol) mono methyl ether (mPEG)) with a molecular weight of 5000 g mol^−1^ was modified with IPTES as described above. This modification retained the same silane concentration as the previously used double modified PEO (10 000 g mol^−1^) but was much closer in molecular weight to PTHF (2900 g mol^−1^), thereby enhancing the mobility of the mPEG chains within the composite slurry and reducing the crystallization of mPEG compared to PEO. The film was prepared in the same manner as PEO_15_PTHF_5_100 with the PEO substituted by the modified mPEG (mPEG_15_PTHF_5_100). This enabled a core‐shell structure of the LLZO particles with mPEG, since the modified mPEG was only able to react with one particle without the possibility of crosslinking, while maintaining a straightforward one‐step slurry roll‐to‐roll process. Here, no competing transport paths compared to the PEO/PTHF mixture were identified based on the DRT analysis (refer to Figure S19, Supporting Information). The mPEG influenced the particle‐PTHF interface as it is an in‐situ formed (partial‐)coating of the LLZO particle acting as an interlayer, which have proven to be beneficial in CPEs.^[^ ^44^ ^]^ Remarkably, we could verify these results of improved ionic conductivity compared to pure PTHF by more than doubling it (*σ* = 3.7 µS cm^−1^ at 60 °C), with the blend exhibiting a significantly more stable ionic conductivity across all temperatures from 20–80 °C. Notably, there was no longer a decline in conductivity below 45 °C, suggesting that the modified blend maintained its efficiency in low‐temperature conditions.

Furthermore, the mixture exhibited an Arrhenius behavior analogous to that of pure PTHF, indicating that the thermal activation energy for ionic transport remains consistent. The *t* ^+^_Li_ for the mPEG/PTHF blend (0.62) was found to be similar to pure PTHF films, indicating that the major contribution of ionic conductivity is through the PTHF polymer matrix and LLZO particles. Hence, gains in ionic conductivity appear to be mainly provided by better interphases through mPEG coating, because a Li^+^‐conducting mPEG phase would severely reduce the lithium transference number as it was observed for PEO_15_PTHF_5_100. These findings highlight the complexity in optimizing electrochemical properties while boosting achievable ionic conductivity, *t* ^+^_Li_, and temperature stability.

Evidently, there is no benefit in direct blending of modified PTHF and PEO due to complete loss of effective lithium conductivity at lower temperatures and only a slight benefit to blending modified PTHF and mPEG. Hence, focus was shifted to using unmodified PEO, in particular Poly(ethylene glycol) dimethyl ether (PEGDME, *M*_n_ = 500 g mol^−1^), which was employed to substitute 25 % of modified PTHF in a PTHF_5_50 film. PTHF_5_50 offered the “best” balance of ionic conductivity and lithium transference number out of all pristine PTHF films. PEGDME is liquid at room temperature and can act as a plasticizer for PTHF and therefore should further enhance the ionic conductivity of PTHF_5_50 while also being able to coordinate Li‐ions itself. As expected, the ionic conductivity of such a plasticized PTHF_5_50 film was boosted by a factor of 2.3 from 6.02 to 13.9 µS cm^−1^ at 60 °C. More importantly, an increase in conductivity at lower temperatures was even more pronounced at 20 °C, as reflected by values going from 0.29·to 1.27 µS cm^−1^, showing a promising increase to room temperature performance. Interestingly, the lithium transference number was almost exactly the median of the transference number of PTHF and PEO with 0.35. This implies a comparatively higher contribution to ionic conductivity of PEGDME than PTHF although it has a significantly lower content in the polymer matrix. Overall, this experiment provided evidence, that there is the potential for significant gains in electrochemical performance through the implementation of plasticizers into the matrix, while also underlining that PTHF still exhibits insufficient performance to be suitable for application as CPEs. This was further corroborated through preliminary plating‐stripping tests where all three PTHF/PEO mixed films failed immediately at a low current density of 0.05 mA cm^−2^. Additional plating‐stripping experiments of PTHF and mixed films are shown in Figures S18–S21 (Supporting Information) for comparison. As the electrochemical performance of the PTHF/PEO mixed films did not warrant an application in battery cells, no mechanical properties were determined.

### 2.5 PEO Film Optimization

**Figure 7:**
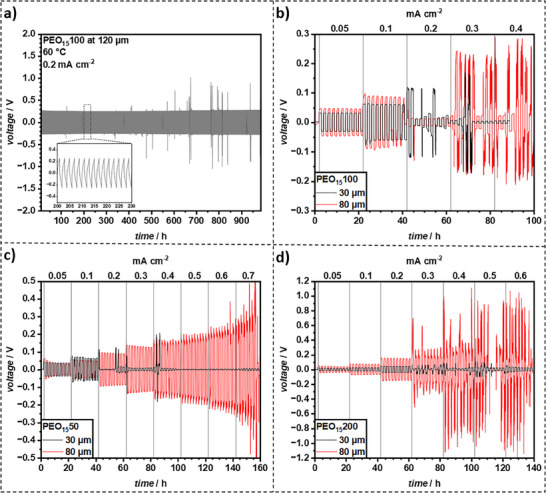
Galvanostatic cycling at 60 °C of a) PEO_15_100 (120 µm thick) at 0.2 mA cm^−2^ and b) PEO_15_100 (30 and 80 µm thick), c) PEO_15_50 (30 and 80 µm thick), and PEO_15_200 (30 and 80 µm thick) with step‐wise increasing current density.

In a preliminary test to assess Li‐metal compatibility of the prepared CPEs, PEO_15_100 with a thickness of 120 µm was cycled at galvanostatic conditions and a current density of up to 0.2 mA cm^−2^ for ≈1000 h at 60 °C (**Figure** **7**a) in CR2032 symmetric Li|PEO_15_100|Li cells. The long‐term cycling test of the 120 µm PEO_15_100 film (Figure 7a) was conducted as an initial, extended assessment of Li‐metal compatibility and SEI stability under prolonged galvanostatic conditions. This thicker film was chosen for this specific test to ensure mechanical robustness over the extended testing period. Over time, the overpotential only slightly increased from 0.25 V (≈200 h) to 0.28 V (≈950 h) indicating high compatibility at Li‐metal interfaces and formation of a stable solid electrolyte interphase (SEI). Overpotential spikes to up to 1 V were observed but aligned with moments when the climate chamber was opened for sample exchanges, leading to unstable temperature profiles as no cell failure was observed for 1000 h. Moreover, cycling under these conditions and current densities exceeded pristine PEO‐LiTFSI SSEs, which typically either failed or showed unfavorable voltage profiles, in this way highlighting the benefits of embedding LLZO into the PEO matrices.^[^ ^45^, ^46^ ^]^ However, due to the resistance resulting from the film thickness, a strong polarization was noted (refer to Figure 7a Inset). Thicker films typically exhibited higher ionic resistance due to the longer diffusion path for lithium ions, while thinner films may offer faster ion conduction but could suffer from lower mechanical stability. Leveraging the outstanding film forming properties and precise manufacturing possibilities of the novel self‐crosslinking CPE system, films of 30 and 80 µm thickness were prepared to assess the influence of the film thickness on the electrochemical performance. The critical current density (CCD) tests on 30 µm and 80 µm films (Figure 7b–d) were subsequently performed to specifically investigate the influence of film thickness and LLZO concentration on the CCD and failure modes, which are critical parameters for practical cell performance.

As aforementioned, PEO films with 50, 100, and 200 % LLZO, showed the best effective Li‐ion conductivity. Hence, these films were evaluated for their critical current density (CCD) through symmetrical plating‐stripping experiments (refer to Figure 7c,d).

For thicker films, a higher overpotential is expected due to the increased resistance associated with greater film thickness. Additionally, thicker films are anticipated to endure higher current densities, primarily because their predominant failure mode in CPEs is dendrite penetration. A dendrite requires more time to penetrate a thicker film, thereby prolonging the time until failure.

The PEO_15_50 film with a thickness of 80 µm exhibited an initial overvoltage of ≈0.065 V, which rapidly decreased to ≈0.034 V at 0.05 mA cm^−2^, suggesting the formation of a stable SEI. This trend of overpotential reduction recurred following each incremental increase in current density up to 0.4 mA cm^−2^, at which point the polarization characteristics of the curve became pronounced. While this progressive behavior could be attributed to successive SEI growth with increasing current density, this hypothesis was not further investigated. In contrast, the PEO_15_50 film with a 30 µm thickness maintained a stable overpotential of ≈0.036 V at 0.05 mA cm^−2^.

For PEO_15_100, the 30 and 80 µm films exhibited overpotentials of ≈0.031 V and ≈0.046 V at 0.05 mA cm^−2^, respectively, which aligned with the expected lower resistance of the thinner film. A similar trend was observed for PEO_15_200, with overpotentials of ≈0.012 V and ≈0.038 V at 0.05 mA cm^−2^ for the 30 and 80 µm films, respectively. In both PEO_15_100 and PEO_15_200, thinner films yielded minimal polarization, whereas the thicker films showed slight polarization as indicated by the shape of the voltage curve. Also, though the reduction in overpotential with cycling was evident, it was notably less pronounced compared to PEO_15_50. Overall, the data suggested that increasing the LLZO concentration results in lower overpotentials in films of similar thickness. However, a direct comparison of the produced films remain challenging due to the intricate effects of interface resistance, interfacial contact, and mechanical properties, all of which are significantly influenced by LLZO concentration.

The results for the determined critical current density (CCD) varied considerably. Both PEO_15_100 films failed rapidly at 0.2 mA cm^−2^, while both PEO_15_200 films sustained a current density of up to 0.3 mA cm^−2^. In contrast, the 30 µm PEO_15_50 film failed at 0.1 mA cm^−2^, whereas the 80 µm film exhibited overvoltage spikes (≈0.39 V) at 0.6 mA cm^−2^ before failing at 0.7 mA cm^−2^. For the 80 µm films, PEO_15_50 (lower LLZO) showing higher CCD than PEO_15_200 (higher LLZO) is counterintuitive if LLZO is assumed to suppress dendrites purely by mechanical blockage. However, PEO_15_50 also exhibited very high strain at break (flexibility), which might accommodate stress better during Li plating/stripping. Higher LLZO content in PEO_15_200 increases stiffness but also reduces ionic conductivity compared to PEO_15_50, which could lead to higher localized current densities and earlier failure despite higher ceramic content. For the 30 µm films, PEO_15_200 (higher LLZO) showing higher CCD than PEO_15_50 (lower LLZO) aligns more with the expectation that ceramic fillers enhance dendrite resistance. Thinner films are generally more susceptible to penetration. The higher ceramic content in PEO_15_200 might offer better mechanical resistance in this thin regime. The interplay is complex, involving mechanical properties (stiffness vs. flexibility), ionic conductivity (affecting homogeneity of current distribution), interfacial resistance, and the stochastic nature of dendrite growth. This complexity and the non‐linear dependencies observed suggest that an optimal LLZO concentration for CCD likely exists and varies with film thickness. For PEO‐based systems the CCD is exceptionally high. Overall, increasing the LLZO concentration increased the overpotential of the thicker films relative to the thinner ones. However, the results regarding CCD remained inconclusive, with no clear trend emerging in relation to film thickness and LLZO concentration.

Alternatively, the CCD for PEO_15_50, PEO_15_100, and PEO_15_200 was determined using Linear Sweep Voltammetry (LSV) for the 80 µm films (Figure S24, Supporting Information). In this assessment, PEO_15_50 and PEO_15_100 exhibited a higher CCD of 0.58 mA cm^−2^, while PEO_15_200 showed a slightly lower CCD of 0.52 mA cm^−2^. It is important to note that CCD determination via LSV generally yields values that are not attainable in Li||Li symmetric cells or full lithium‐metal batteries. Consequently, CCD values obtained from LSV measurements tend to be overestimated.

#### 2.5.1 NMC Compatibility of PEO CPEs

The selection strategy for PEO compositions in full cells was as follows: PEO_15_100 was chosen for initial NMC C‐rate tests as it showed a good balance of ionic conductivity across temperatures and a relatively high *t* ^+^_Li_, making it a reasonable starting point for full cell evaluation. PEO_15_50 and PEO_15_200 were subsequently chosen for NMC cycle life tests to explore the impact of lower (50%) and higher (200%) LLZO content on CEI stability with the challenging NMC cathode, considering their different mechanical properties (PEO_15_50 high flexibility, PEO_15_200 higher stiffness) and CCD behaviors observed in symmetric cells.

**Figure 8:**
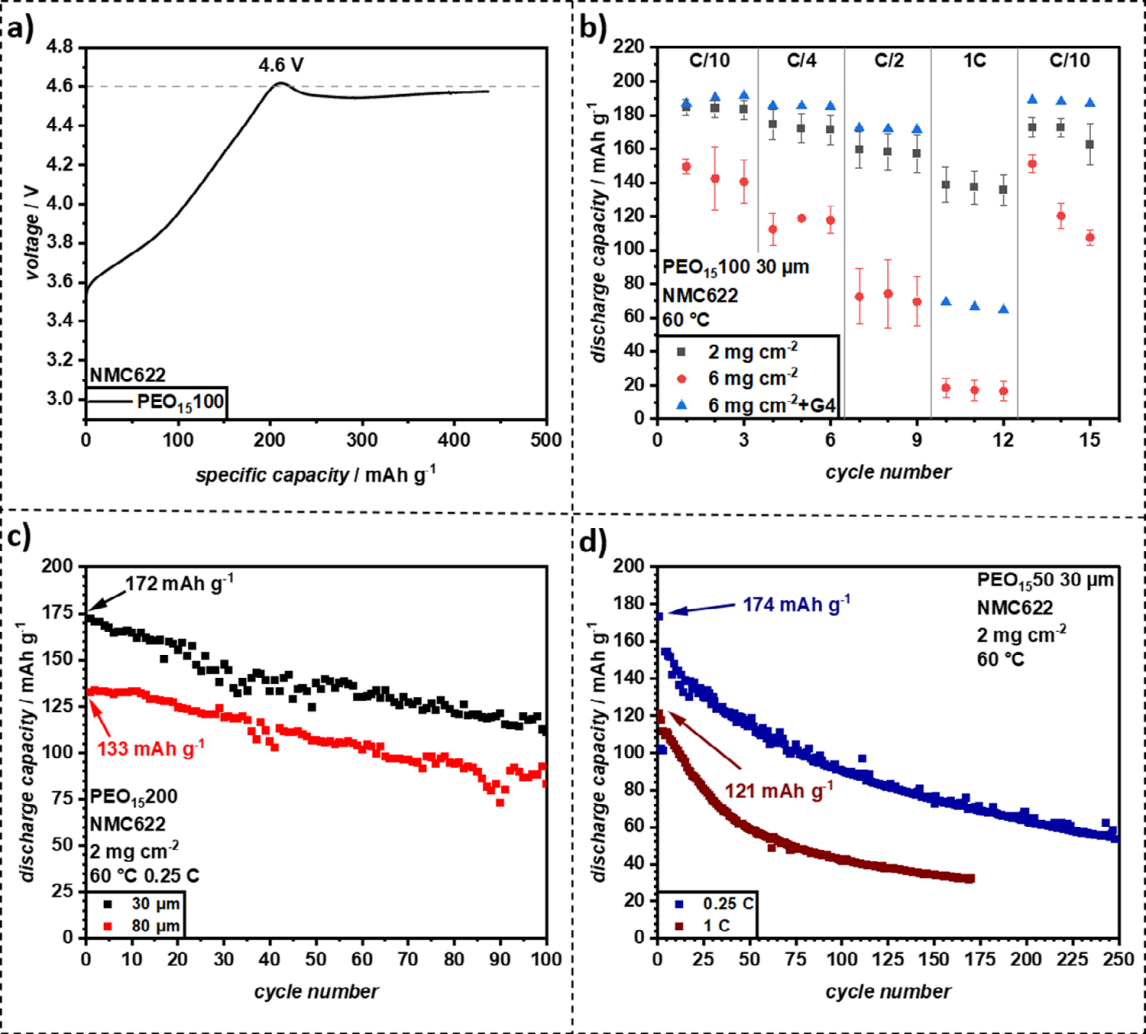
a) Electrochemical stability of PEO_15_100 against NMC_622_ determined by galvanostatic overcharging (0.1 C). b) C‐rate capability tests of PEO_15_100 (30 µm) with NMC_622_ with a mass loading of 2, 6, and 6 mg cm^−2^ with the addition of 5 µl of 1 m LiTFSI in tetraethylene glycol dimethyl ether (G4) at 60 °C. c) Constant current cycling of PEO_15_200 at 0.25 C of Li|PEO_15_200|NMC_622_ with 2 mg cm^−2^ mass loading at 60 °C. d) Constant current cycling of PEO_15_50 (30 µm) at 0.25 C and 1 C of Li|PEO_15_200|NMC_622_ with 2 mg cm^‑2^ mass loading at 60 °C.

To evaluate the electrochemical stability of PEO‐LLZO CPEs, galvanostatic overcharging at 0.1 C was performed against LiNiMnCoO_2_ (Ni:Mn:Co 6:2:2) NMC_622_ as a high‐voltage cathode (refer to **Figure** **8**a). A voltage plateau at 4.6 V indicated the onset of oxidative decomposition. Given that NMC_622_ typically charges at 4.3 V vs. Li|Li⁺, the PEO‐LLZO CPEs were expected to be compatible with NMC_622_ cathodes, leading to the assembly of Li|PEO_15_100|NMC_622_ CR2032 cells for compatibility testing. A 30 µm film was invoked to minimize internal resistance in initial C‐rate tests (see Figure 8b). Cells with cathode active mass loadings of 2 mg cm^−2^ (self‐made) and 6 mg cm^−2^ (CustomCells) were evaluated, as the solid‐state electrolytes typically struggle with high‐mass‐loading porous cathodes due to limited contacts.

After three charge‐discharge cycles at 0.05 C, discharge capacities were 184.5 mAh g^−1^ (2 mg cm^−2^) and 149.5 mAh g^−1^ (6 mg cm^−2^). While the former cells met the rated 175.0 mAh g^−1^ capacity, the latter cells expectedly exhibited significant capacity losses. To improve contacts, 5 µL of 1 m LiTFSI in tetraethylene glycol dimethyl ether (G4) was infiltrated as a catholyte into the 6 mg cm^−2^ CAM cathodes. G4, a compound structurally similar to PEO‐LiTFSI but liquid at room temperature, enhanced wettability, increased discharge capacity to 186.9 mAh g^−1^ which exceeded the 2 mg cm^−2^ cell, likely due to measurement error of the cell dimensions.

C‐rate tests were conducted at 0.1 C, 0.25 C, 0.5 C, 1 C, and back to 0.1 C. At 2 mg cm^−2^, 75% of initial capacity was retained at 1 C, comparable to similar systems.^[^ ^6^ ^]^ At 6 mg cm^−2^, rate capability declined due to increased current density. The addition of G4 boosted performance, maintaining 37% of initial capacity at 1 C versus 12% for pristine NMC_622_. Furthermore, pristine 6 mg cm^−2^ CAM mass loading NMC_622_ showed irreversible capacity losses, which G4 mitigated.

In view of long‐term cycling, Li|PEO_15_50/200|NMC_622_ cells with 2 mg cm^−2^ CAM are shown exemplary. At lower mass loading, reduced current stress allowed better assessment of cathode‐electrolyte interface (CEI) stability (refer to Figure 8c,d). The 30 µm PEO_15_200 film afforded an initial discharge capacity of 172.6 mAh g^−1^, close to the theoretical capacity of 175.5 mAh g^−1^, whereas the 80 µm film achieved 133.3 mAh g^−1^. Both showed strong capacity fluctuations, likely reflecting parasitic oxidative decomposition at the cathode interfaces. After 100 cycles, the capacity retention was 65% (30 µm) and 62% (80 µm). Comparatively, a similar silane‐mPEG‐coated LLZO PEO‐LiTFSI system by Ma et al. cycled at 0.2 C in a NMC_811_||Li cells retained 82% of the initial 156 mAh g^−1^ capacity after 100 cycles, indicating improvement potential.^[^ ^6^ ^]^

Further cycling tests at 0.25 and 1 C showed instability of the PEO‐LLZO cathode interfaces (see Figure 8d). At 0.25 C, the initial discharge capacity was 174.2 mAh g^−1^, decreasing to 52% after 100 cycles. At 1 C, initial capacity dropped to 121.3 mAh g^−1^, with retention falling to 35%. Interestingly, discharge curves were less noisy at 1 C, but capacity fade was more severe than for PEO_15_200 (30 µm), reinforcing CEI instability as a key limitation.

Note that the rapid capacity fading of PEO‐based electrolytes operated with high‐voltage cathodes (>4 V vs Li|Li⁺) is well‐documented and attributed to the oxidative decomposition of PEO, even catalyzed by the NMC surfaces.^[^ ^47^ ^]^ Thus, mitigation strategies, such as Al_2_O_3_ coatings on NMC, have been developed. However, since commercially available coated NMC cathodes were not accessible, further analysis of PEO‐CPEs with NMC cathodes was considered not fruitful and thus was not pursued at this point.

#### 2.5.2 LFP Compatibility of PEO CPEs

**Figure 9:**
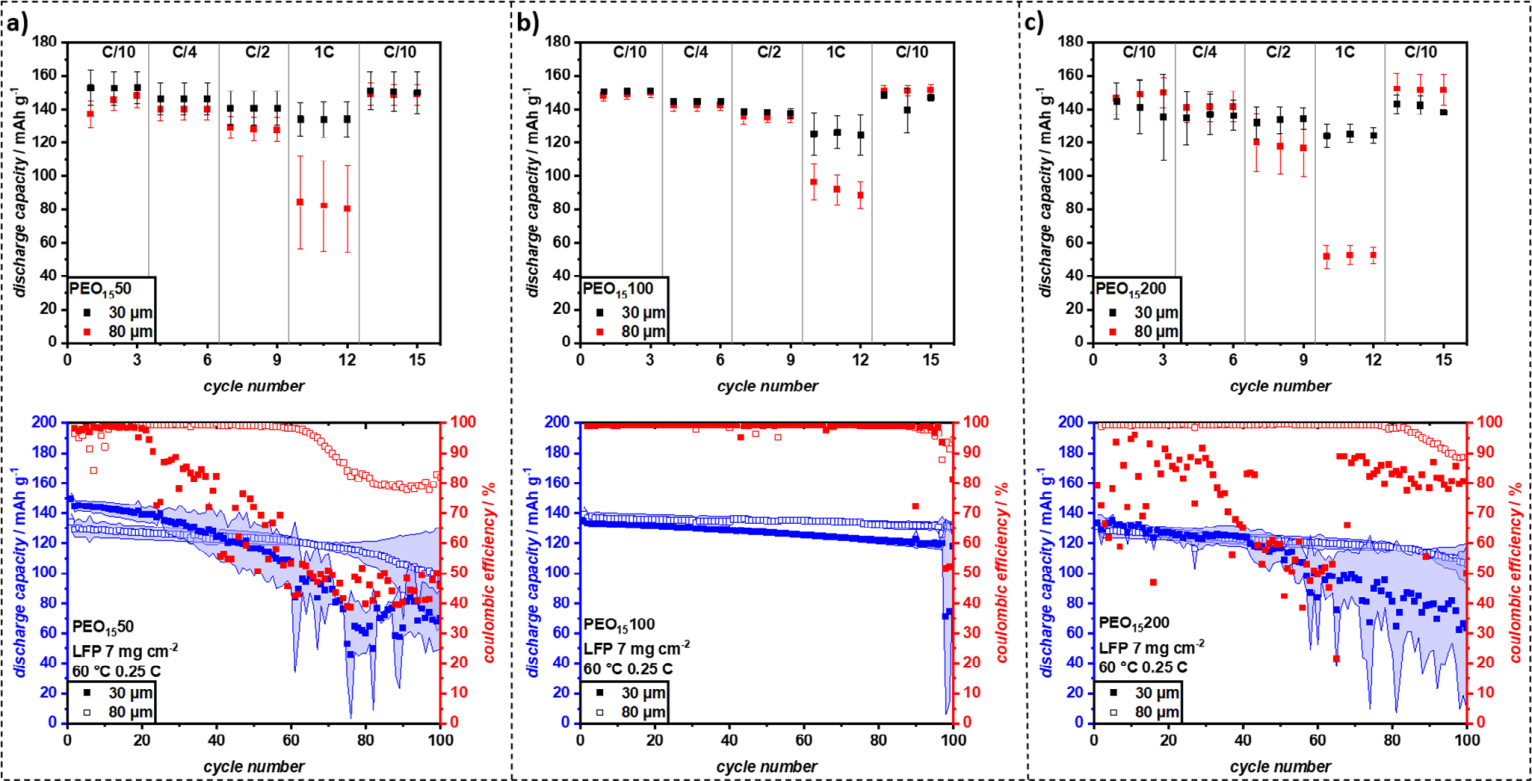
C‐rate capability tests and consecutive constant current cycling with LFP with a mass loading of 7 mg cm^−2^ with the addition of 5 µl of 1 m LiTFSI in G4 at 60 °C for a) PEO_15_50, b) PEO_15_100, and c) PEO_15_200. The shaded area in the bottom row represents the error in capacity of the average of three cells, while the symbols indicate the capacity of one representative cell.

Given the better stability expected with LFP, we expanded the testing to PEO_15_50, PEO_15_100, and PEO_15_200 to comprehensively assess performance (C‐rate and cycling) across a wider range of LLZO concentrations and film thicknesses. This allowed for a more thorough investigation of the impact of LLZO content with a more stable cathode. To address the oxidative stability issues with high‐voltage cathodes, NMC cathodes were replaced with commercial LiFePO_4_ (LFP) cathodes (maximum voltage of 4.0 V, circumventing PEO decomposition) with mass loadings of 7 mg cm^−2^ (specific capacity: 160 mAh g^−1^, supplied by CustomCells). C‐rate capability tests, incorporating also G4 additive, were repeated. Again, PEO_15_50, PEO_15_100, and PEO_15_200 films were utilized with 30 and 80 µm thicknesses in Li|CPE|LFP full cells (refer to **Figure** **9** top).

For PEO_15_50 and PEO_15_100, the 30 µm films exhibited higher discharge capacities at all C‐rates, whereas PEO_15_200 showed higher discharge capacities for the 80 µm film at 0.1 C and 0.25 C. However, due to measurement variability, no explicit correlations could be established. On average, the 30 µm films across all LLZO concentrations demonstrated a high capacity retention at 1 C (83–88%). In contrast, the 80 µm films showed wider variation, with 57% retention for PEO_15_50, 64% for PEO_15_100, and 35% for PEO_15_200. Despite this, these retention values surpassed those of NMC_622_ cathodes (34% with 6 mg cm^−2^ + G4), even with higher mass loadings.

Notably, the increased mass loading resulted in a current density of 0.15 mA cm^−2^ at 0.1 C, approaching the limit observed in plating‐stripping tests, while at 1 C, the current density reached 1.5 mA cm^−2^, which was well beyond stable cycling conditions for PEO‐based systems.^[^ ^48^ ^]^ Indeed, the CCD values observed in symmetric Li||Li cells (e.g., PEO_15_100 failing at 0.2 mA cm^−2^, PEO_15_50 30 µm failing at 0.1 mA cm^−2^) are lower than this. This apparent discrepancy is a known phenomenon and can be attributed to several factors including different cell configurations (symmetric cells are often more prone to rapid dendrite‐induced failure), differences in SEI formation in the presence of a cathode (which does not involve Li plating/stripping), the effect of stack pressure and contact quality (especially with the G4 additive wetting the cathode), and the difference between short‐term C‐rate tests versus sustained current application in CCD tests. Despite this, the thin films performed remarkably well in the C‐rate tests, most likely due to their low internal resistance.

Following the rate capability tests, the cells underwent constant current cycling at 0.25 C and 60 °C (see Figure 9, bottom). At 0.25 C, the applied current density (0.375 mA cm^−2^) exceeded the CCD of all films previously determined in plating‐stripping tests (max. 0.3 mA cm^−2^). However, the stable cycling observed in several films suggests that the actual CCD of the CPEs may be higher than indicated by plating‐stripping experiments as was previously assumed.

Despite the demanding C‐rate capability tests, the 30 µm films retained high initial discharge capacities of 152.2, 135.1, and 130.9 mAh g^−1^ for PEO_15_50, PEO_15_100, and PEO_15_200, respectively. Similarly, the 80 µm films exhibited slightly lower or similar discharge capacities of 138.4, 140.5, and 133.2 mAh g^−1^ for the same compositions.

During constant cycling, the 30 µm films degraded faster than the 80 µm films, likely due to dendrite penetration. PEO_15_50 (30 µm) exhibited pronounced capacity fading and a noisy voltage profile, reaching 80% state of health (SOH) after only 58 cycles. PEO_15_200 (30 µm) performed slightly better, maintaining 80% SOH for 88 cycles, while PEO_15_100 (30 µm) demonstrated the most stable cycling, with minimal noise, a Coulombic efficiency (CE) of 99.7%, and 80% SOH after 136 cycles.

Increasing the film thickness to 80 µm significantly enhanced the cycling stability. While initial discharge capacities were slightly lower, voltage profiles were smoother, and the cycle life until reaching 80% SOH was extended to 130, 140, and 200 cycles for PEO_15_50, PEO_15_100, and PEO_15_200, respectively (Figure S25, Supporting Information). This trend indicated that higher LLZO content improves cycling stability at increased film thickness. Additionally, PEO_15_100 (80 µm) exhibited an increased CE of 99.8%. It was observed that PEO_15_200 showed the lowest initial overpotentials in symmetric cells at 0.05 mA cm^−2^ for both 30 µm and 80 µm films. However, in LFP full cells (Figure 9), particularly the 30 µm films, PEO_15_100 (and PEO_15_50) showed slightly better capacity retention at 1C compared to PEO_15_200. Possible reasons for this include differences in overall cell impedance (PEO_15_100 has slightly higher ionic conductivity than PEO_15_200 which might be critical at high C‐rates), potentially different interfacial contact with the cathode where PEO_15_100 might offer a better balance of conductivity and mechanical compliance, and the fact that symmetric cell polarization was measured at a much lower current density (0.05 mA cm^−2^) than the 1C rate in full cells (1.5 mA cm^−2^), where dominant resistance contributions can change. Given the high current densities and relatively low film thicknesses, these results highlight a substantial improvement of the crosslinked PEO‐LLZO CPEs over similar systems.

For comparison, Cai et al. developed a polymer‐in‐ceramic electrolyte by infiltrating a Li_6.4_La_3_Zr_2_Al_0.2_O_12_ (3D LLZAO) framework with PEO‐LiTFSI.^[^ ^49^ ^]^ Their electrolyte maintained 80% SOH for ≈100 cycles at 0.2 C in an LFP||Li cell, but utilized a significantly lower mass loading (1.5 mg cm^−2^), resulting in a lower current density. Moreover, their fabrication process involved a non‐scalable, energy‐intensive sponge template infiltration. In contrast, self‐crosslinking PEO‐LLZO CPEs developed in our study demonstrated superior electrochemical performance with a significantly simpler synthesis.

## 3 Conclusion

The solvent‐free synthesis approach presented in this work offers a straightforward and scalable route to producing high‐performance composite polymer electrolytes suitable for operation in solid‐state lithium metal batteries. The ability to mix different polymers and introduce functional additives provides exceptional flexibility in solid polymer electrolyte design, allowing for targeted optimization of electrochemical and mechanical properties. Moreover, homogeneous dispersion of ceramic fillers circumvents agglomeration, thus ensuring consistent ionic conductivity and mechanical integrity.

Roll‐to‐roll processing allows for manufacture of films as thin as 30 µm, bestowing minimal internal resistances and improved charge carrier transport. The self‐crosslinking mechanism enhances mechanical stability, preventing cracking or tearing even at conventionally difficult to manufacture thicknesses.

Notably, the hybrid CPEs demonstrate outstanding compatibility with higher‐mass‐loading cathodes, enabling superior cycling performance and high energy density. At 60 °C, the optimized PEO‐based CPEs exhibit an ionic conductivity of up to 0.27 mS cm^−1^. Additionally, the LLZO boosts the Li^+^ transference number compared to pristine PEO‐based systems, significantly improving ion transport, while reducing polarization during cycling, and increasing oxidative stability (4.6 V vs Li|Li^+^). These enhancements contribute to the superior cycling performance observed in LFP|CPE|Li cells, where the best‐performing compositions maintained 80% capacity retention over 200 cycles at 0.25 C.

The broader significance of this work lies in several key aspects. First, the solvent‐free, scalable synthesis method is a crucial advantage for industrial relevance, moving away from lab‐scale complexities. Second, the demonstrated tunability, exemplified by the easy variation of polymers (PEO and PTHF), fillers (LLZO content), and additives, allows for systematic tailoring of electrolyte properties, even if PTHF systems did not ultimately match PEO performance, they showcased the versatility of the synthesis platform. Thirdly, the capability to produce thin films (down to ≈30 µm) is critical for reducing internal resistance and enhancing the energy density of practical SSB cells. The high performance achieved with PEO‐LLZO systems, particularly the long cycle life with high‐mass‐loading LFP cathodes is competitive and highlights the potential of this approach. While full cell testing of PTHF‐based systems was not pursued due to their lower ionic conductivity, the detailed characterization provided for PTHF offers valuable insights for future material design using this synthesis approach.

The ability to explicitly tailor electrolyte composition and processing conditions renders the introduced approach highly adaptable for future advancements in polymer‐based solid‐state battery technology. Indeed, this innovative platform represents a significant step toward the commercialization of solid‐state batteries, combining straightforward fabrication, customizable material properties, and superior achievable electrochemical performance of the cells.

Detailed post‐mortem interfacial studies, such as X‐ray photoelectron spectroscopy (XPS), would be a valuable direction for future work to further elucidate degradation mechanisms and guide rational interface engineering, complementing the current electrochemical stability assessments derived from long‐term cycling and high Coulombic efficiencies.

## Conflict of Interest

Daniel Döpping, Dominik Voll, and Patrick Théato have a patent application filed for the fabrication of the CPE films outlined in this article.

## Supporting information



Supporting Information

## Data Availability

The data that support the findings of this study are available in the supplementary material of this article.
