# Supplementary material for: Versatile Solvent‐Free Synthesis of Composite Polymer Electrolytes for Thin High‐Performance Solid‐State Lithium Metal Batteries
Source: Small. 2025 Jul 30;21(37):e04166. doi: 10.1002/smll.202504166 (PMC12444833; doi:10.1002/smll.202504166)
Supplement: Supplementary file 1 — Supporting Information [file SMLL-21-e04166-s001.docx]

**Supporting Information**

**Versatile Solvent-free Synthesis of Composite Polymer Electrolytes for Thin High Performance Solid-State Lithium Metal Batteries**

Daniel Döpping^a^, Annika Buchheit^b^, Xiaochen Liu^c^, Anika Goecke^a^, Alexander P. Grimm^d^, Dominik Voll^a^, Manfred Wilhelm^a^, Martin Finsterbusch^c^, Martin Winter^b,d^, Gunther Brunklaus^b^, and Patrick Théato*^a,e^

a. Institute for Chemical Technology and Polymer Chemistry (ITCP), Karlsruhe Institute of Technology (KIT), Engesserstraße 18, D-76131 Karlsruhe, Germany

b. Institute of Energy Materials and Devices (IMD-4: Helmholtz-Institute Münster, Ionics in Energy Storage), Forschungszentrum Jülich GmbH, Corrensstraße 48, D-48149 Münster, Germany

c. Institute of Energy Materials and Devices: Materials Synthesis and Processing (IMD-2) Forschungszentrum Jülich GmbH, Wilhelm-Johnen-Straße, D-52425 Jülich, Germany

d. MEET Battery Research Center, Institute of Physical Chemistry, University of Münster, Corrensstraße 46, D-48149 Münster, Germany

e. Soft Matter Synthesis Laboratory, Institute for Biological Interfaces III, Karlsruhe Institute of Technology (KIT), Hermann-von-Helmholtz-Platz 1, D-76344 Eggenstein-Leopoldshafen, Germany

The experimental data used in this study is available under DOI: https://dx.doi.org/10.22000/bfcgd6zhx6uu861n

Contents

[Instruments 3](#_Toc200110902)

[Synthesis 7](#_Toc200110903)

[Materials 7](#_Toc200110904)

[LLZO Synthesis 7](#_Toc200110905)

[Polymer Modification 7](#_Toc200110906)

[Film Preparation 8](#_Toc200110907)

[Experimental Data 11](#_Toc200110908)

[Ionic Conductivity 11](#_Toc200110909)

[Oscillatory Rheology and Tensile Strength tests 12](#_Toc200110910)

[Differential Scanning Calorimetry 13](#_Toc200110911)

[Thermogravimetric analysis 14](#_Toc200110912)

[Distribution of Relaxation Time (DRT) analysis 17](#_Toc200110913)

[Constant Current Cycling 26](#_Toc200110914)

[Linear Sweep Voltammetry 29](#_Toc200110915)

[Analytics of synthesized LLZO 30](#_Toc200110916)

[X-Ray Diffraction (XRD) 30](#_Toc200110917)

[Particle Size Distribution (PSD) 31](#_Toc200110918)

[Nyquist Plot of pristine LLZO 31](#_Toc200110919)

[Inductively Coupled Mass Spectrometry (ICP-MS) of LLZO 32](#_Toc200110920)

[Analytics of Polymers 32](#_Toc200110921)

[Size Exclusion Chromatography (SEC) 32](#_Toc200110922)

[Nuclear Magnetic Resonance (NMR) 36](#_Toc200110923)

[Attenuated Total Reflection Fourier-Transform Infrared Spectroscopy (ATR-IR) 39](#_Toc200110924)

[References 40](#_Toc200110925)

# Instruments

**Oscillatory Rheology**

Rheological measurements were conducted using a strain-controlled ARES G2 rheometer (TA Instruments) through small amplitude oscillatory shear experiments. The measurements were performed in the linear regime at frequencies ranging from 0.1 to 100 rad s^-1^ at a temperature of 25 °C, under a nitrogen atmosphere. A 13 mm parallel plate geometry was used, with a strain amplitude of γ_o_ = 0.1 %. The samples were prepared from films obtained via the self-crosslinking membrane preparation method, each having a diameter of 13 mm and a thickness of 0.2 mm.

**Tensile Strength**

Hegewald und Peschke Meß-und Prüftechnik GmbH, Universalprüfmaschine inspekt table. Equipped with a 1 kN force transducer. Measurements were performed under ambient conditions with punched out bones (DIN 53504 S3) of 2.5 cm length and 5 mm width. Films had a thickness of 200 µm.

**Scanning electron microscope (SEM) and energy-dispersive X-ray spectroscopy (EDX)**

FEI Helios G4 equipped with a Schottky Thermal Field Emission Cathode. The energy range is 20 eV to 30 keV for secondary and backscattered electron imaging, with beam current ranges of 0.8 pA - 100 nA. The Bruker XFlash 6|60 EDXS detector (60mm² SDD, slew AP3.3 window) is used for elemental analysis, the Everhart-Thornley (ETD) detector for secondary and backscattered electron detection, and the In-Chamber Electron (ICE) detector for capturing emitted electrons. Additional detectors include a Through-Lens Detector (TLD), Mirror Detector, and In-Column Detector (ICD) for high-quality SEM imaging, as well as the STEM detector with BF, ADF, and HAADF segments for detailed structural analysis. The system also includes an EBSD (Electron Backscatter Diffraction) and TKD (Transmission Kikuchi Diffraction) detector head for crystal structure analysis, texture, and phase analysis.

Samples were sputtered with gold after being glued with adhesive graphite tabs on an aluminum sample holder.

Scanning electron microscopy imaging of cross-sections was done using a *Quanta 200 F* from *FEI* with an accelerating voltage of 15 kV. Samples were sputtered with gold for 240 seconds prior to measurement.

**Nuclear Magnetic Resonance (NMR)**

The ^1^H-NMR and ^13^C-NMR spectra were acquired using a Bruker Ascend III 400 MHz spectrometer, with operating frequencies of 400 MHz for proton and 101 MHz for carbon. All samples were dissolved in deuterated solvents, and the chemical shifts are referenced to the residual solvent signals.

**Attenuated Total Reflection Fourier-Transform Infrared Spectroscopy (ATR-IR)**

ATR-IR spectra were recorded on a Bruker Alpha II IR from 400 – 4000 cm^-1^ at 22 °C.

**Thermogravimetric Analysis (TGA)**

All thermogravimetric analysis measurements were performed on a TGA 5500 (TA Instruments) in Platinum HT pans under nitrogen atmosphere. Samples are equilibrated at 30 °C and heated to 800 °C at a heating rate of 10 K min^-1^.

**Differential scanning calorimetry (DSC)**

Differential scanning calorimetry was measured on a Netzsch DSC Polyma 214 equipped with liquid nitrogen cooling. Samples of 2-10 mg were measured in aluminum pan with a pierced lid in two heating cycles. The measurements were usually performed from ‑150 to 160 °C at a heating and cooling rate of 10 K min^-1^.

**Size exclusion chromatography (SEC)**

Size exclusion chromatography was performed in tetrahydrofuran (THF) using a Tosoh Bioscience HLC-8320GPC EcoSEC system, which was equipped with an autosampler, three PSS SDV columns (5 μm, 100 Å, 1000 Å, 100000 Å; 8 × 300 mm), and both UV and differential refractive index (RI) detectors. The system operated at a temperature of 35 °C with a flow rate of 1 mL min^-1^. The system was calibrated with ReadyCal standards, using poly(methyl methacrylate) (PMMA) standards with molecular weights ranging from 800 to 2200000 g mol^-1^, polystyrene (PS) standards from 370 to 2520000 g mol^-1^, and poly(ethylene oxide) standards from 238 to 217000 g mol^-1^. Usually, 50 μL of a filtered 2.0 mg mL^‑1^ sample solution was injected onto the columns. The eluent contains 0.55 g BHT per 2.5 l HPLC grade THF.

**Electrochemical Measurements**

**Electrochemical Impedance Spectroscopy (EIS)**

Electrochemical impedance spectroscopy was used for determination of the ionic conductivity. Therefore, CR2032 coin cells in symmetrical arrangement with stainless steel discs as electrodes with the samples in between were measured on a Multi Autolab M204 with a FRA32M-module (Deutsche Metrohm) and the data were collected with the software NOVA 2.1.4 (Metrohm Autolab B.V.). Logarithmic frequency scans of 50 steps from 1 to 10^6^ Hz with an alternating voltage of 40 mV were performed at all temperature settings 20 to 80 °C, adjusted via climatic chamber (MK 053, Binder GmbH, Germany), temperature fluctuations did not exceed ±0.02 K). Ionic conductivity was calculated according to:

$$\sigma=\frac{1}{R}*\frac{d}{\pi*\pi^{2}}$$

whereas, σ is the ionic conductivity, *R* the ohmic resistance, *d* the sample thickness and *r* the radius of the sample.

**Transference Number**

Transference numbers were measured according to Bruce-Vincent method.^[1,2]^ Therefore, symmetrical CR2032 coin cells with lithium electrodes with the electrolyte sandwiched in between were built. The measurements were performed at 60 °C in a climatic chamber (MK 053, Binder GmbH, Germany), temperature fluctuations did not exceed ±0.02 K. Complete transference experiments including polarization and intermediate impedance measurements were performed on an Multi Autolab M204 equipped with FRA32M-module (Deutsche Metrohm) and the data were collected with NOVA software 2.1.4 (Metrohm Autolab B.V.). The cells were equilibrated for 24 h, meanwhile the impedance of the polymer/lithium boundary was monitored in order to ensure the formation of a stable SEI. For determination of transference numbers, a constant DC voltage of 10 mV (ΔV) was applied to the cell and the time dependent current was monitored, the initial current is denoted as *I*_0_ and the final steady state current is denoted as *I*_ss_ in the following. The cell impedance was analyzed before and after each single DC polarization step by AC impedance measurements. Therefore, logarithmic frequency scans of 50 steps from 1 to 10^6^ Hz with an alternating voltage of 10 mV were performed. The ohmic part of the electrolyte impedance and the lithium/electrolyte interface impedance is denoted as *R*_el,0_ and *R_Li,_*_ss_ for the sample before polarization and after polarization in steady state, respectively. Calculation of transference numbers were done according to:

$$t_{Li}^{+}=\frac{R_{el,0}*I_{ss}}{\Delta V-R_{Li,ss}*I_{ss}}$$

DRT analysis was performed on RelaxIS 3 (version 3.0.20.16, rhd instruments GmbH & Co. KG) software.

**LSV/CV**

LSV and CV experiments were carried out with an Autolab PGSTAT302 N (Metrohm) potentiostat at 60 °C, data were collected with the software NOVA 2.1.4 (Metrohm Autolab B.V.). Samples were measured in a three-electrode setup in Swagelok cells, whereas Lithium served as the counter and reference electrode. For reductive scan, Nickel was used as working electrode, for oxidative scan Platinum serves as the working electrode respectively. Reductive scan was performed between OCV and -0.4 V vs. Li│Li+ or until lithium deposition, oxidative scan was performed between OCV and 7 V vs. Li│Li+ or the current exceeds 10^-5^ A.

**Cell Building**

NMC_622_ cathodes (2 mg cm^-2^) were made according to literature.^[3]^

NMC_622_ electrodes consisting of 90 wt.% (1.854 g) NMC_622_-Powder (BASF TODA Battery Materials LLC), 7 wt.% (0.144 g) Carbon Black (Super C65, Imerys Graphite & Carbon) and 3 wt.% (0.062 g) PVdF (Solef® 5130, Solvay) were produced by first dissolving PVdF in 4 mL (3.883 g) NMP (99.5%, Sigma Aldrich) using an ARM-310 ThinkyMixer (THINKY) for 20 Minutes at 2000 RPM. Carbon Black and NMC622 were subsequently added to the mixture and homogenized with the ThinkyMixer for 20 min. at 2000 RPM and 10 min. at 300 RPM to get rid of any remaining air bubbles within the mixture. The slurry was cast onto previously cleaned aluminum foil (20 µm, Evonik Industries) by using a doctor blade (ZUA 2000 Universal Applicator, ZEHNTER GmbH) with a wet coating thickness of 40 - 50 µm and an Automatic Film Applicator (1133 N, Sheen Instruments) with a speed of 50 mm s^−1^. The electrode sheets were dried in an oven (Binder GmbH) at 80 °C for 2 hours before drying under reduced pressure (10^-3^ mbar) over night at 110 °C. This step was followed by calendaring to a thickness of ~ 34 - 38 µm and punching into circular electrodes using a Hohsen electrode puncher (Hohsen Corp.) with a diameter of Ø = 12 mm and further dried again over night at reduced pressure (10^-5^ mbar) before being used for cell production. The produced cathodes are corresponding to ~2 mg cm^-2^ CAM.

NMC_622_ (6 mg cm^-2^) and LFP (7 mg cm^-2^) cathode sheets with a mass loading of 1 mAh cm^-2^ were purchased from CustomCells GmbH, pre-dried for 12 h at 110 °C and punched to circular discs of 12 mm. 50 µm thick lithium metal foil (Honjo) was used as anode material and punched to circular discs of 14 mm. Cells were built by stacking cathode and anode with a hybrid film of 16 mm in diameter in between in CR2032 coin cells. All cells were built in a dryroom (dewpoint < -60°C).

**Constant Current Cycling**

All constant current cycling experiments were conducted using a coin cell-type two-electrode setup with a Maccor® Series 4000 battery test system. The cells were conditioned at 60°C in a climate chamber (Memmert) and equilibrated for 12 h before each test. Lithium plating and stripping experiments were carried out in symmetrical lithium cells, where an initial conditioning phase at 0.05 mA cm^-2^ for 10 cycles was performed. Subsequently, lithium was plated and stripped at 0.2 mA cm^-2^ either for one hour per cycle over 500 cycles equivalent to 1000 hours or until failure.

For plating and stripping experiments with alternating current densities, an initial 0.05 mA cm^-2^ for 10 cycles were conducted before gradually increasing the current density every 10 cycles in increments of 0.1 mA cm^-2^, until either a short circuit occurred, or safety limits were reached.

Galvanostatic overcharging (upper voltage limit: 6 V) was performed at 18.0 mA g⁻¹ (~0.1 C) with NMC_622_ cathodes. The voltage plateau observed during testing was used to identify the onset of oxidative decomposition of the polymer electrolyte against the cathode materials.

Full cell cycling experiments were conducted between 3.0 V - 4.3 V vs. Li|Li^+^ for NMC_622_ and 2.8 - 4.0 V vs. Li|Li^+^ for LFP at a charge rate of 0.25 C. For rate performance evaluations, the charge and discharge rate were varied between 0.1 C and 1 C in steps of 0.25 C.

Cells were acclimated at OCV for 12 h at 60 °C. Subsequently for the three forming cycles, cells were charged with constant current - constant voltage protocol with constant voltage for 30 min or < 0.05 C. Discharge cycles were performed under constant current.

# Synthesis

## Materials

Poly(ethylene oxide) (10000 g mol^-1^, Sigma Aldrich), Poly(tetrahydrofuran) (2900 g mol^-1^, Sigma Aldrich), 3‑(Triethoxysilyl)propyl isocyanate (95%, TCI Chemicals), Tetrahydrofuran (99%, VWR), Diethyl ether (99%, VWR), Bis(trifluoromethane)sulfonimide lithium salt (LiTFSI, 99.95% trace metal basis, Sigma Aldrich), Mylar foil (PPI-SP 914 colorless, 100 µm, PPI Adhesive Products ), Polyethylene glycol dimethyl ether (500 g mol^-1^, Sigma Aldrich), LiOH (99%, Applichem), ZrO_2_ (99.7%, Treibacher), La_2_O_3_ (99%, Merck, pre-dried at 900 °C for 10 h), Al_2_O_3_ (99.82%, Inframat), Ta_2_O_5_ (99.95%, Treibacher), PVdF (Solef® 5130, Solvay), NMP (99.5%, Sigma Aldrich), Carbon Black (Super C65, Imerys Graphite & Carbon), aluminum foil (20 µm, Evonik Industries)

## LLZO Synthesis

Li_6.45_Al_0.05_La_3_Zr_1.6_Ta_0.4_O_12_ was prepared by solid-state synthesis. First, stoichiometric amounts of LiOH (Applichem, 99%), ZrO_2_ (Treibacher, 99.7%), La_2_O_3_ (Merck, 99%, pre-dried at 900 °C for 10 h), Al_2_O_3_ (Inframat, 99.82%), and Ta_2_O_5_ (Treibacher, 99.95%) were used. A 10 wt% excess of LiOH was used to compensate for lithium evaporation during sintering. The mixture was thoroughly mixed (Retsch, 200 M) and pressed into pellets, which were then calcined at 850 ◦C for 20 h. The obtained pellets were crushed and dry milled (Retsch, PM400) in 250 mL tungsten carbide jars with 20 mm tungsten carbide milling balls at 200 rpm, and the powder was sieved with a 50 µm sieve (Fritsch, Ana lysette). The obtained powder was thoroughly mixed and pressed into particles again and then calcined at 1000 ◦C for 20 h. After that, the particles were crushed and dry-milled again. The obtained powder was stored in a glove box.

## Polymer Modification

Figure S1 General modification procedure of hydroxy-terminated polymers with IPTES.

As an example, the synthesis of IPTES-PEO is described. The other polymers were synthesized in the same manner with 2 equivalents of 3-(triethoxysilyl)propyl isocyanate per 1 equivalent of hydroxy groups.

PEO (*M*_n_=10,000 g·mol^-1^, Sigma Aldrich; 50.0 g, 5.00 mmol, 1.00 eq.) and 3-(triethoxysilyl)propyl isocyanate (95%, abcr; 4.95 g, 20.0 mmol, 4.00 eq.) are added to a 100 mL flask and purged with nitrogen for 15 min. The flask is heated to 75 °C for 24 h and stirred at a low rate since the molten polymer is highly viscous. THF (99%, VWR) is added after cooling the flask below 60 °C. Precipitated from THF in ice-cold Et_2_O (99%, VWR) three times. The modified polymer is dried in a vacuum oven overnight at 35 °C 600 mbar.

## Film Preparation

The polymers, LiTFSI, LLZO, and additives are stored in a glovebox. 1 g of polymer, LiTFSI according to the [O]:[Li] ratio (in the index of the film name), and LLZO in ratios of 25, 50, 100, 200 or 400 wt% in regard to the pure polymer without LiTFSI are weighed in. Additives are weighed in according to the stated amount. All components are taken out of the glovebox and mixed in a mortar until a homogeneous slurry is obtained. The slurry is sandwiched between two Mylar® foils and calendared through a manual roll-to-roll press (Xiamen Tmax Battery Equipments Limited, Manual Roller Press Machine 100 mm diameter and 200 mm width roll dimensions with 0.2 µm tolerance on roll circumference.) with adjustable slit width. The Mylar® foil has a thickness of 100 µm so the slit width was adjusted to 200+x with x being the desired film thickness. All roll-pressed slurries were dried under ambient conditions for 24 h to obtain a flexible cross-linked film. Subsequently, the films are dried twice. First, in a Büchi Schlenkline (<5·10^-3^ mbar) at 60 °C and afterwards on a Highcube (<10^-6^ mbar) at 60 °C. Films are stored in dry room with a dew point of -60 °C.

It is important to note that the simplified naming scheme of the films (e.g., PEO_15_100 having 100 % of the mass of LLZO added in regard to PEO mass) does not correctly reflect the actual wt% of the components in the slurry/CPE as the weight of LiTFSI is omitted. However, to correctly assess whether a CPE is classified as a ceramic-in-polymer (CIP, ceramic < 50 wt%) or a polymer-in-ceramic (PIC, ceramic > 50 wt%) the ceramics content is of great importance. Although the classification of CIP vs. PIC does not directly indicate the interplay of ionic transport properties of polymer/interface Li-ion transport vs. active particle Li-ion transport, it shows the dominating phase present in the CPE which must be considered when interpreting experimental results. Hence, in Table S1 a comprehensive list of classifications of all PTHF and PEO CPEs is given with their respective LLZO wt%.

Albeit PEO and PTHF being different polymers and therefore having slightly different densities and amounts of LiTFSI added, in both cases all films with 100 % and less of LLZO added are considered CIP. 200 % and higher means LLZO had > 50 wt% of total mass and these systems were considered as PIC. Since additives were added in the mg scale, their influence on the overall mass distribution was negligible and did not change the classification.

The thinnest achieved film thickness with high enough mechanical strength to enable cell preparation was 16 µm. However, since not all combinations could be roll-pressed to that thickness, 30 µm and 80 µm were generally used as the standard film thickness unless otherwise stated.

Table S1 Actual wt% of LLZO in the slurry of manufactured PEO and PTHF CPEs with the corresponding classification into ceramic-in-polymer (CIP) or polymer-in-ceramic (PIC) systems.

| **Film** | **wt% of LLZO** | **Classification** |
| --- | --- | --- |
| PEO_15_ |  |  |
| 25 | 15.0 | CIP |
| 50 | 26.1 |  |
| 100 | 41.4 |  |
| 200 | 58.6 | PIC |
| 400 | 73.8 |  |
| PTHF_5_ |  |  |
| 25 | 12.9 | CIP |
| 50 | 22.9 |  |
| 100 | 37.3 |  |
| 200 | 54.3 | PIC |
| 400 | 70.4 |  |

During the synthesis of the CPE films incorporating various polymers, oxide particles, and concentrations, several key observations were made during the film fabrication process. However, these findings could not be reliably quantified due to the influence of numerous variables, some of which were difficult to control, affecting the overall process.

The rate at which the slurry upon mixing solidified was highly dependent on:

Variable (faster > slower crosslinking condition)

1. polymer (hygroscopic > hydrophobic)
2. particle concentration (higher > lower)
3. particle surface (oxides > phosphates)
4. temperature (higher > lower)
5. humidity (higher > lower)
6. viscosity (higher > lower)

Overall, the slurry crosslinking time varied between 3 minutes and several days, depending on the CPE components used. In some cases, particularly at high oxide concentrations (> 200 %), the addition of small amounts of ethyl acetate as a plasticizer was required. Ethyl acetate reduced the viscosity, enhanced the mixability of components, and delayed the onset of crosslinking, thereby facilitating controlled roll pressing. All in all, the influence on the crosslinking rate by these variables highly indicated that a moisture catalyzed reaction between the alkoxy silane end-groups and hydroxy-groups at the particle surface were the main crosslinking contributor and not homocondensation of alkoxysilanes. This hypothesis was supported through rheological and EDX measurements.


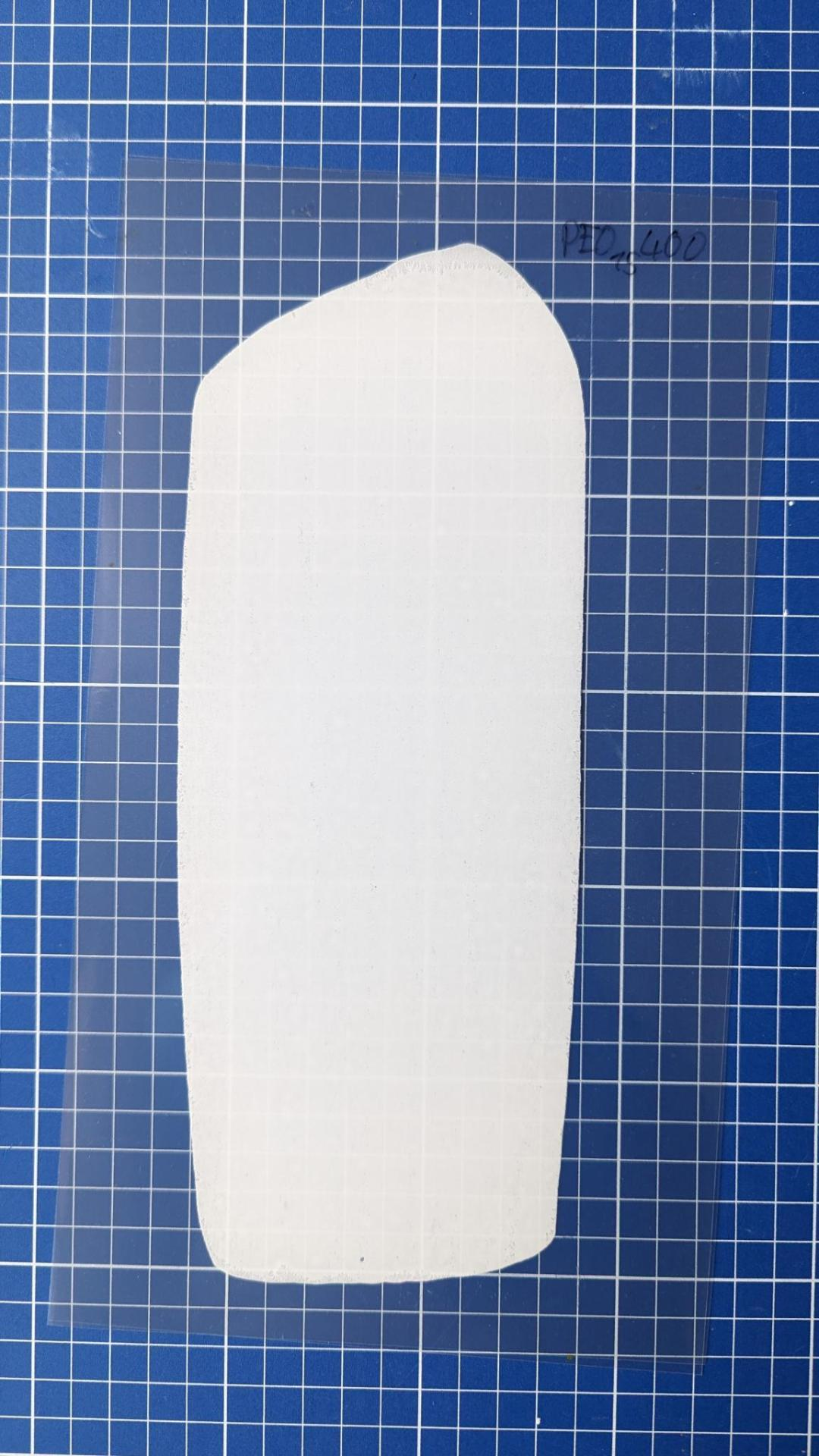


Figure S2 A homogeneous PEO_15_400 film after roll-to-roll pressing in between two Mylar® foils (blue squares have a size of 1x1 cm)

# Experimental Data

## Ionic Conductivity


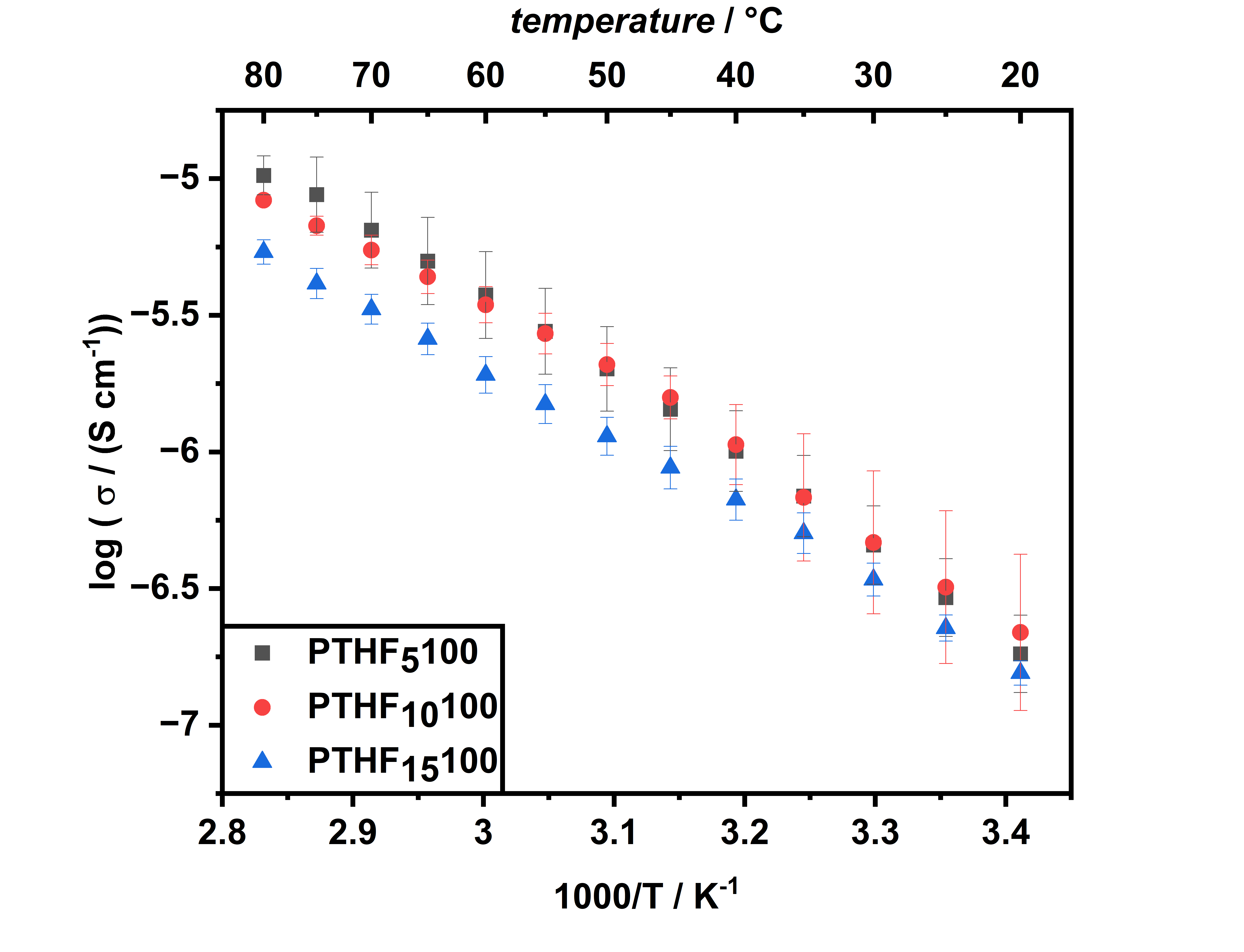


Figure S3 Ionic conductivity of PTHF_5_100 at 5, 10, and 15 [O]:[Li] ratio from 20-80 °C.

## Oscillatory Rheology and Tensile Strength tests


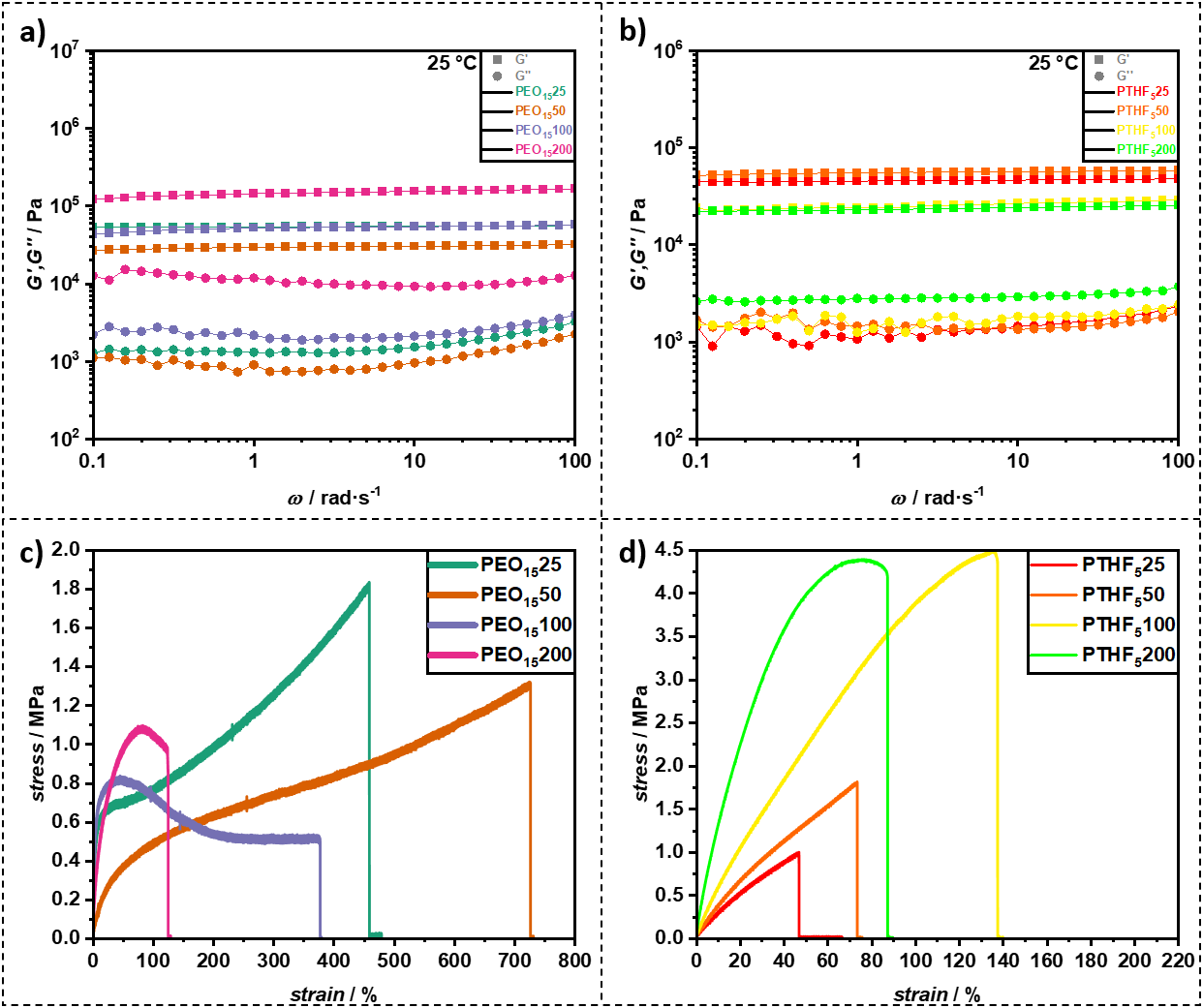


Figure S4 Frequency sweep of PEO_15_25-200 (a) and PTHF_5_25-200 (b) from 100 to 0.1 rad s^-1^ at 25 °C. Tensile strength of PEO_15_25-200 (c) and PTHF_5_25-200 (d) at 25 °C.

## Differential Scanning Calorimetry

Figure S5 DSC thermograms of PEO_15_25-400 from -150 - 150 °C at a heating rate of 10 K min^-1^. Depicted is the 2^nd^ heat run.

Figure S6 DSC thermograms of PTHF_5_25-400 from -150 - 150 °C at a heating rate of 10 K min^-1^. Depicted is the 2^nd^ heat run.

Figure S7 DSC thermograms of mixed PEO/PTHF films from -150 - 150 °C at a heating rate of 10 K min^-1^. Depicted is the 2^nd^ heat run.

## Thermogravimetric analysis

Figure S8 TGA measurements of PEO_15_25-400 at a heating rate of 10 K min^-1^ from 30 - 800 °C.

All PEO-based CPEs have a degradation temperature of close to 360 °C.

Figure S9 TGA measurements of PTHF_5_25-400 at a heating rate of 10 K min^-1^ from 30 - 800 °C.

The decomposition of PTHF-based CPEs varies with LLZO content. Generally, decomposition is observed at 230 °C and 330 °C.

Figure S10 TGA measurements of mixed PTHF films at a heating rate of 10 K min^-1^ from 30 - 800 °C.

The decomposition of mixed PTHF films depends on the PEO phase. All films show decomposition at 360 °C while the PEGDME containing film also shows a mass loss at 240 °C due to the evaporation of PEGDME (Bp ~240 °C).

## Distribution of Relaxation Time (DRT) analysis

**PEO CPEs**

Figure S11 Development of areal resistance of PEO CPEs over 20 h in symmetrical Li||Li cells.

Figure S12 Nyquist plot of PEO CPEs after 20 h in symmetrical Li||Li cells.

Figure S13 DRT of PEO CPEs after 20 h in symmetrical Li||Li cells.

**PTHF CPEs**

Figure S14 Development of areal resistance of PTHF CPEs over 20 h in symmetrical Li||Li cells.

Figure S15 Nyquist plot of PTHF CPEs after 20 h in symmetrical Li||Li cells.

Figure S16 DRT of PTHF CPEs after 20 h in symmetrical Li||Li cells.

**Mixed CPEs**

Figure S17 Development of areal resistance of mixed CPEs over 20 h in symmetrical Li||Li cells. PEO_15_100 and PTHF_5_100 are added as a reference.

Figure S18 Nyquist plot of mixed CPEs after 20 h in symmetrical Li||Li cells. PEO_15_100 and PTHF_5_100 are added as a reference.

Figure S19 DRT of mixed CPEs after 20 h in symmetrical Li||Li cells. PEO_15_100 and PTHF_5_100 are added as a reference.

## Constant Current Cycling

Figure S20 Symmetrical Li||Li plating-stripping experiments of PTHF_5_50-200-LLZO films (30 µm) with increasing current density after 10 cycles at 60 °C.

PTHF_5_50-200 exhibit stable Li-plating at 0.05 and 0.1 mA cm^-2^ but immediately fail at 0.2 mA cm^-2^ (PTHF_5_100/200) and 0.3 mA cm^-2^ (PTHF_5_50). However, PTHF_5_50 shows overpotentials of > 1.0 V at 0.2 mA cm^-2^ and strong polarization with unstable voltage profiles. Overall, PTHF CPEs show slight polarization at all tested current densities and Li-ion transport seems too low for application in Li-metal batteries.

Figure S21 Symmetrical Li||Li plating-stripping experiments of PTHF_5_25/400-LLZO films (30 µm) with increasing current density after 10 cycles at 60 °C.

No stable plating-stripping behavior is observed for PTHF_5_25/400 as the films immediately fail.


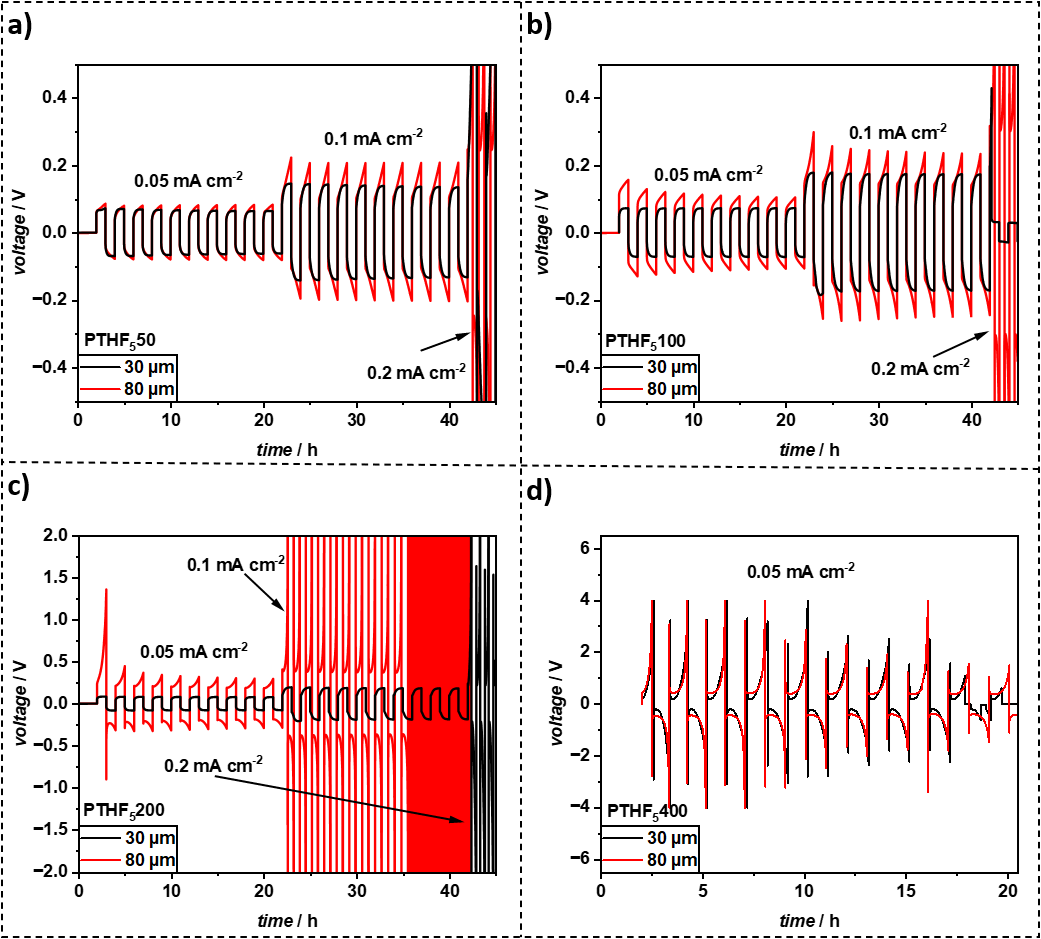


Figure S22 Symmetrical Li||Li plating-stripping experiments of PTHF_5_50-400-LLZO films (30 µm and 80 µm) with increasing current density after 10 cycles at 60 °C.

Figure S23 Symmetrical Li||Li plating-stripping experiments of all PEO_15_PTHF_5_LLZO (30 µm and 80 µm) with increasing current density after 10 cycles at 60 °C.

Mixed PTHF films do not exhibit any stable Li-plating behavior.

## Linear Sweep Voltammetry

Figure S24 Determination of critical current density of PEO_15_50, PEO_15_100, and PEO_15_200 (80 µm) by LSV at 2.0·10^-5^ V s^-1^.


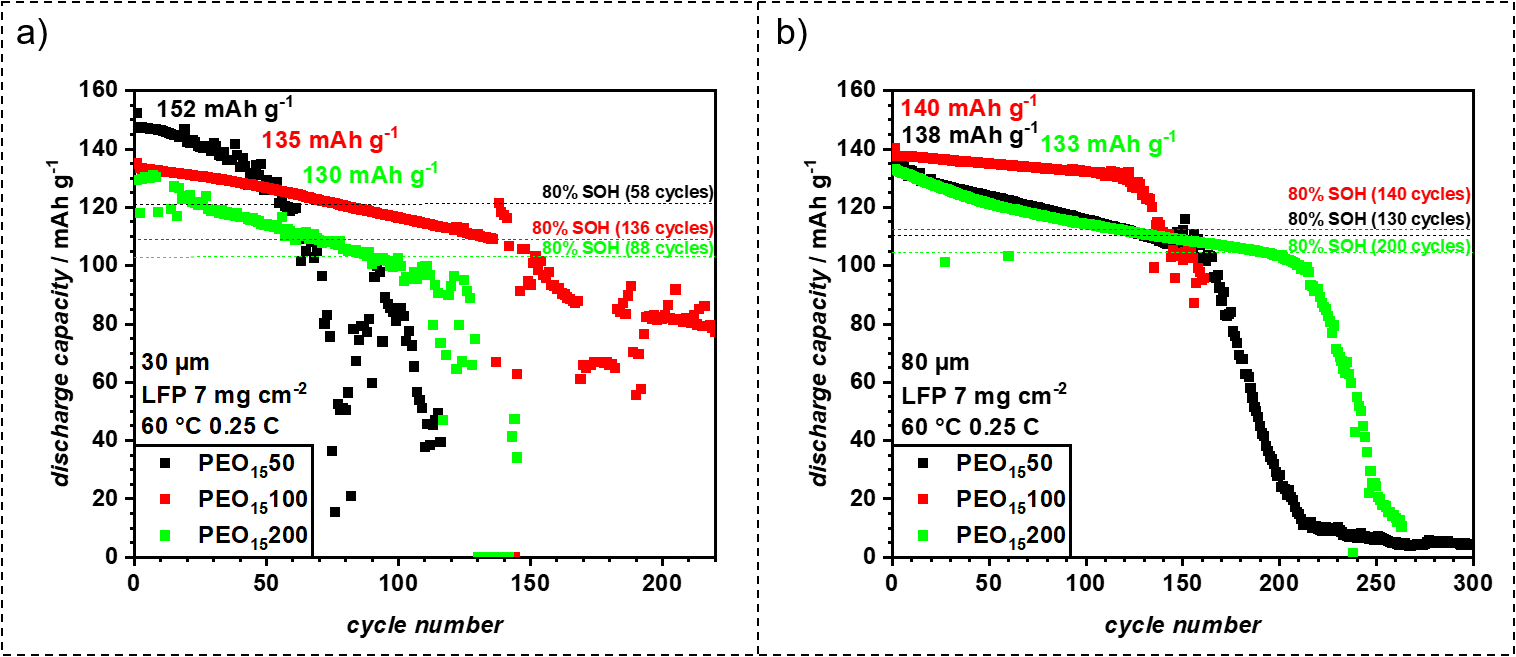


Figure S25 Constant current cycling of PEO_15_50, PEO_15_100, and PEO_15_200 (30 µm (a) and 80 µm (b)) at 0.25 C of Li|CPE|LFP with 7 mg cm^‑2^ mass loading at 60 °C and their respective initial discharge capacity. Depicted are the best performing cells out of three for each LLZO concentration. In general, 80 µm films exhibit longer cycling stability than 30 µm films. Coulombic efficiency is omitted for clarity.

## Analytics of synthesized LLZO

### X-Ray Diffraction (XRD)


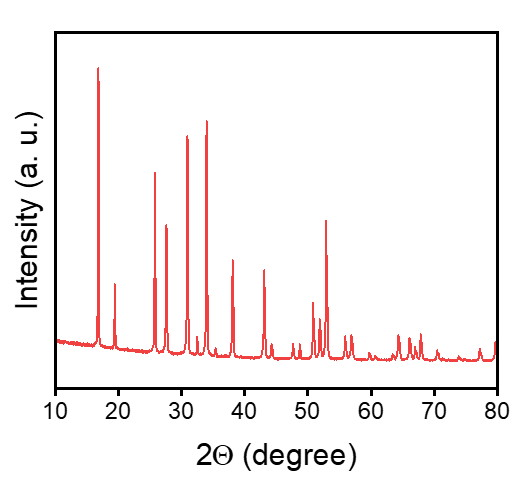


Figure S26 The XRD patterns of synthesized LLZO.

### Particle Size Distribution (PSD)


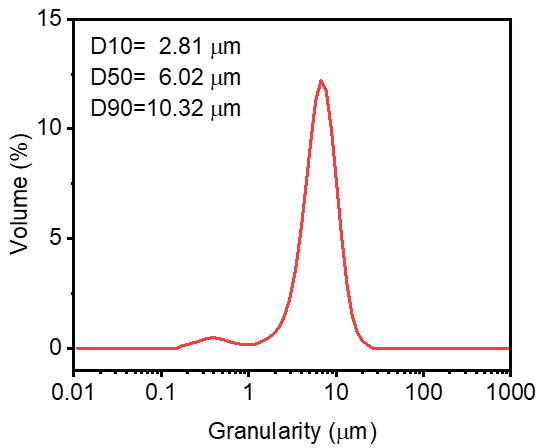


Figure S 27 Particle size distribution (PSD) of LLZO particles.

### Nyquist Plot of pristine LLZO

Figure S28 EIS spectra of LLZO block cell at room temperature

### Inductively Coupled Mass Spectrometry (ICP-MS) of LLZO

Table S2 Inductively coupled plasma mass spectrometry (ICP) of LLZO,

| **Element** | **MW%** | **M / g mol^-1^** | **N / mol** | **factor** | **n (ICP results)** | **n (target)** |
| --- | --- | --- | --- | --- | --- | --- |
| Li | 5.3 | 6.94 | 0.764 | 12.496438 | 6.73 | 6.45 |
| La | 48.1 | 138.91 | 0.346 |  | 3.05 | 3 |
| Zr | 14.6 | 91.22 | 0.160 |  | 1.41 | 1.6 |
| Ta | 8.1 | 180.95 | 0.045 |  | 0.39 | 0.4 |
| Al | 0.245 | 26.98 | 0.009 |  | 0.08 | 0.05 |

## Analytics of Polymers

### Size Exclusion Chromatography (SEC)

Figure S29 THF-SEC of unmodified PEO as calibrated via a PMMA standard.

Figure S30 THF-SEC of IPTES-modified PEO as calibrated via a PMMA standard.

Figure S31 THF-SEC of unmodified mPEG as calibrated via a PMMA standard.

Figure S32 THF-SEC of IPÜTES-modified mPEG as calibrated via a PMMA standard.

Figure S33 THF-SEC of unmodified PTHF as calibrated via a PMMA standard.

Figure S34 THF-SEC of IPTES-modified PTHF as calibrated via a PMMA standard.

### Nuclear Magnetic Resonance (NMR)

Figure S35 ^1^H-NMR spectrum of IPTES-modified PEO in CDCl_3_ with proton assignment.

**^1^H NMR (400 MHz, CDCl_3_)** *δ* / ppm: 4.49 - 4.07 (m, 4H), 3.86 - 3.73 (m, 12H), 3.72 - 3.38 (m, 200H), 3.49 - 3.38 (m, 4H), 1.66 - 1.50 (m, 4H), 1.28 - 1.09 (m, 18H), 0.68 - 0.48 (m, 4H).

Due to the high concentration of ethylene glycol units an accurate measurement of proton ratios for ethylene -C*H*_2_- is not possible.

Figure S36 ^1^H-NMR spectrum of IPTES-modified mPEG5000 in CDCl_3_.

**^1^H NMR (400 MHz, CDCl_3_)** *δ* / ppm: 4.01 (t, *J* = 4.8 Hz, 2H), 3.63 (qd, *J* = 7.0, 2.1 Hz, 6H), 3.52 - 3.34 (m, 300H), 3.29 (q, *J* = 7.0 Hz, 2H), 3.19 (s, 3H), 2.97 (q, *J* = 6.5 Hz, 2H), 1.51 - 1.30 (m, 2H), 1.12 - 0.88 (m, 9H), 0.54 - 0.30 (m, 2H).

Figure S37 ^1^H-NMR spectrum of IPTES-modified PTHF in CDCl_3_ with proton assignment. b is hidden underneath the g peak.

**^1^H NMR (400 MHz, CDCl_3_)** *δ* / ppm: 3.80 (q, *J* = 7.0 Hz, 4H), 3.47 - 3.32 (m, 50H), 3.15 (q, *J* = 7.0 Hz, 4H), 1.63 - 1.53 (m, 50H), 1.29 - 0.95 (m, 18H), 0.73 - 0.50 (m, 4H).

### Attenuated Total Reflection Fourier-Transform Infrared Spectroscopy (ATR-IR)

Figure S38 ATR-IR of IPTES-modified PEO.

Figure S39 ATR-IR of IPTES-modified PTHF.

# References

[1] P. G. Bruce, C. A. Vincent, *Journal of Electroanalytical Chemistry and Interfacial Electrochemistry* **1987**, *225*, 1.

[2] J. Evans, C. A. Vincent, P. G. Bruce, *Polymer* **1987**, *28*, 2324.

[3] F. Scharf, A. Krude, P. Lennartz, M. Clausnitzer, G. Shukla, A. Buchheit, F. Kempe, D. Diddens, P. Glomb, M. M. Mitchell, T. Danner, A. Heuer, A. Latz, M. Winter, G. Brunklaus, *Small* **2024**, *20*, 2404537.
